# *Fusarium oxysporum* mediates systems metabolic reprogramming of chickpea roots as revealed by a combination of proteomics and metabolomics

**DOI:** 10.1111/pbi.12522

**Published:** 2016-01-23

**Authors:** Yashwant Kumar, Limin Zhang, Priyabrata Panigrahi, Bhushan B. Dholakia, Veena Dewangan, Sachin G. Chavan, Shrikant M. Kunjir, Xiangyu Wu, Ning Li, Pattuparambil R. Rajmohanan, Narendra Y. Kadoo, Ashok P. Giri, Huiru Tang, Vidya S. Gupta

**Affiliations:** ^1^ Division of Biochemical Sciences CSIR‐National Chemical Laboratory Pune India; ^2^ Key Laboratory of Magnetic Resonance in Biological Systems National Centre for Magnetic Resonance in Wuhan Wuhan Institute of Physics and Mathematics Chinese Academy of Sciences Wuhan China; ^3^ Central NMR Facility CSIR‐National Chemical Laboratory Pune India; ^4^ State Key Laboratory of Genetic Engineering, Metabolomics and Systems Biology Laboratory School of Life Sciences Fudan University Shanghai China

**Keywords:** Chickpea, *Fusarium oxysporum*, plant–pathogen interaction, proteomics, metabolomics, NMR

## Abstract

Molecular changes elicited by plants in response to fungal attack and how this affects plant–pathogen interaction, including susceptibility or resistance, remain elusive. We studied the dynamics in root metabolism during compatible and incompatible interactions between chickpea and *Fusarium oxysporum* f. sp. *ciceri* (Foc), using quantitative label‐free proteomics and NMR‐based metabolomics. Results demonstrated differential expression of proteins and metabolites upon Foc inoculations in the resistant plants compared with the susceptible ones. Additionally, expression analysis of candidate genes supported the proteomic and metabolic variations in the chickpea roots upon Foc inoculation. In particular, we found that the resistant plants revealed significant increase in the carbon and nitrogen metabolism; generation of reactive oxygen species (ROS), lignification and phytoalexins. The levels of some of the pathogenesis‐related proteins were significantly higher upon Foc inoculation in the resistant plant. Interestingly, results also exhibited the crucial role of altered Yang cycle, which contributed in different methylation reactions and unfolded protein response in the chickpea roots against Foc. Overall, the observed modulations in the metabolic flux as outcome of several orchestrated molecular events are determinant of plant's role in chickpea–Foc interactions.

## Introduction

Chickpea (*Cicer arietinum* L.) is the second most widely grown legume in the world. As a top producer, India contributes about 90% of global chickpea production (http://faostat.fao.org/site/339/default.aspx). Chickpea is mainly used as a primary vegetarian source of human dietary protein and, thus, is of significance to food and nutritional security in the developing world. However, due to widespread occurrence of fungal pathogens, such as *Fusarium oxysporum* and *Ascochyta rabei*, the yield of chickpea has been constrained in spite of successive efforts of national and international breeding programmes. Annual yield losses due to wilt disease alone have been estimated to range from 10 to 90% (Anjaiah *et al*., 2003; Jimenez‐Diaz *et al*., 1989). Wilt is caused by eight races of *Fusarium oxysporum* f. sp. *ciceri* (Foc) affecting all the major chickpea growing areas (Gurjar *et al*., 2009). Foc infects roots and clogs the xylem, resulting in the obstruction of nutrient supply as wilting progresses, ultimately leading to plant death. This fungus can survive for many years in soil even without its host and, hence, poses a serious challenge for disease management (Haware *et al*., 1996).

Many studies have been carried out to identify the molecular basis of Foc resistance or susceptibility in chickpea using various approaches including gene mapping, candidate gene identification, differential expression and biochemical analysis postfungal infection (Ashraf *et al*., 2009; Giri *et al*., 1998; Gowda *et al*., 2009; Gupta *et al*., 2010, 2013a; Gurjar *et al*., 2012; Nimbalkar *et al*., 2006). However, genomic scale dynamics of plant–fungus interaction is poorly understood. Further, due to variations in chromosomal rearrangements between nonmodel and model plants, differences in the plant–pathogen interaction with respect to signalling and immunity events are also expected and evinced (Gupta *et al*., 2013a). Therefore, studies on nonmodel plants using unbiased modern high‐throughput technologies are required to improve our knowledge of the plant–fungus interactions (Kushalappa and Gunnaiah, 2013; Mehta *et al*., 2008). In recent years, a combination of metabolomics and gene‐expression analysis has been employed to understand such interactions (Liu *et al*., 2010) and the effects of gene manipulation on the systems metabolic changes of *Fusarium graminearum* (Chen *et al*., 2011). A few studies using functional genomics (Cho *et al*., 2012; Golkari *et al*., 2007), proteomics (Lee *et al*., 2009; Yang *et al*., 2010) and metabolomics (Bollina *et al*., 2011; Kumaraswamy *et al*., 2011) approaches have also been reported on plant–pathogen systems of Fusarium head‐blight and cereal crops.

As plant–fungus interactions are complex in nature and factors leading to resistance or susceptibility in plant remain largely obscure, the objective of current study was to assess overall modulations in the levels of proteins and metabolites in chickpea roots upon Foc inoculation. Time series profiling of proteome and metabolome of Foc‐inoculated resistant and susceptible chickpea roots was performed using label‐free quantitative proteomics and untargeted ^1^H‐NMR metabolomics. We observed highly orchestrated response with significant modulation in various metabolic processes. The results described here thus improve our fundamental knowledge of molecular dynamics associated with the chickpea–Foc interaction and potentially useful in designing strategies against wilt disease in chickpea.

## Results

### Protein identification and quantification in Foc‐inoculated chickpea roots

**Figure 1:**
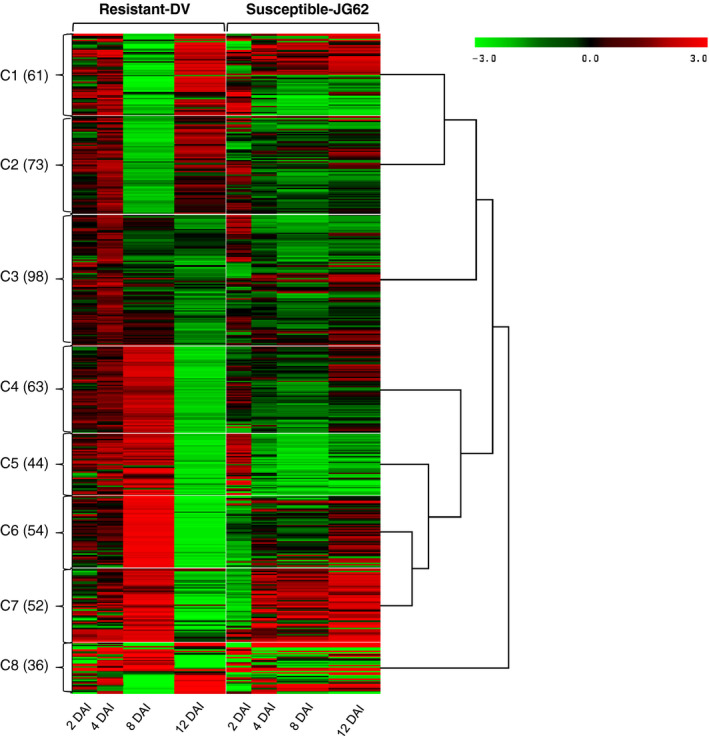
Clusters (C1 to C8) of 481 differential expressed proteins in chickpea root. For each protein, the ratio of Log_2_‐normalized expression of Foc inoculated with its respective control at various stages (2 to 12 DAI) and represented by a colour, according to the colour scale at the top. The number of proteins in a given cluster with similar expression trend is indicated in parentheses.

Two days after inoculation (DAI) with Foc, the susceptible chickpea cultivar (JG62) showed yellowing phenotype followed by drooping of leaves that finally lead to complete wilting by 12 DAI. Whereas, Foc‐inoculated resistant cultivar (Digvijay‐DV) and the mock‐inoculated wilt susceptible and resistant cultivars remained healthy throughout the experimental period. The JG62 plant could not sustain the fungal invasion beyond 12 DAI, while DV plants remained unaffected (Figure S1). Based on these phenotypes, 2 and 4 DAI were considered as early stage, while 8 and 12 DAI were considered as late stage. We conducted high‐throughput label‐free quantitative proteomics analysis with Foc‐ and mock‐inoculated chickpea root tissues at various time points from 2 to 12 DAI. This analysis identified a total of 811 proteins (Table S1a) from which fungal proteins were excluded in further analysis (Lee *et al*., 2009). From these, 481 had statistically significant differential expression (*P *< 0.05 and fold change >1.2) across cultivars and over the course of infection (Tables S1b and c). The ratio of normalized intensity of proteins from the inoculated samples vis‐a‐vis respective controls revealed increased or decreased expression in the Foc‐inoculated roots. The log_2_‐transformed values of differentially expressed proteins were clustered using SplineCluster (Heard *et al*., 2006), which generated eight clusters using a prior precision of 1 × 10^−4^ (Figure 1).

### Protein expression patterns in Foc‐inoculated resistant and susceptible cultivars

The potential biological function for each cluster was deduced based on gene ontology enrichment analysis using BiNGO. Cluster 1 (C1) had 61 proteins enriched for isoflavonoids biosynthesis and response to oxidative stress (Figure 1), while there were 73 proteins enriched in lignin biosynthesis, s‐adenosyl methionine biosynthetic process and glycolytic process in C2. Majority of proteins from C1 and C2 revealed higher expression at all the stages except 8 DAI in DV compared to JG62. The C3 cluster with 98 proteins was enriched for stress response, malate metabolism, oxidation–reduction process and glycolytic pathway. These proteins had increased expression at early stages which decreased at later stages in DV. Total 63 proteins from C4 cluster were enriched for the response to misfolded proteins, microtubule polymerization processes, osmotic stress response, proteosome core complex assembly and gluconeogenesis. The C5 cluster had 44 proteins enriched for protein degradation through ubiquitin, ATP biosynthesis, photorespiration and active proton (H^+^) transport. Proteins from C4 and C5 clusters showed general trend of higher expression in all the stages except 12 DAI in DV. On the contrary, JG62 showed lower expression in majority of these proteins at later stages (Figure 1). Total 54 proteins had high expression in C6 at all the stages except 12 DAI in DV compared to JG62 and did not show enrichment to any specific process through BiNGO. The C7 cluster had 52 proteins that were enriched in defence response and oxidative stress response. These proteins displayed overall contrasting expression patterns in DV and JG62 at early (2 DAI) and late (12 DAI) stages. The 36 proteins from C8 cluster were enriched in response to abiotic stress, fatty acid biosynthesis and nucleosome assembly processes.

### Foc induced quantitative variation in proteins from important metabolic pathways

**Figure 2:**
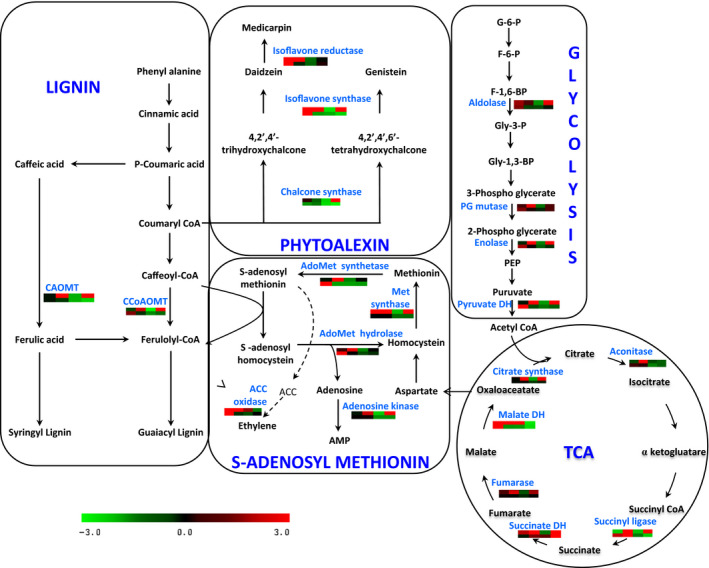
Interconnection between various metabolic processes in chickpea–Foc interaction. Each graph represents differential expression (fold change) pattern according to the colour scale; columns represent the four stages (2 to 12 DAI) after Foc inoculation. The first row represents the resistant cultivar, while the second row represents the susceptible cultivar.

Upon Foc inoculation, we observed a complex response in chickpea from interconnected metabolic pathways including primary amino acid metabolism, glycolysis/gluconeogenesis, TCA cycle, phenylpropanoid pathway and increased lignification (Figure 2). Other cellular processes altered during Foc infection included unfolded protein response (UPR) and defence‐related proteins. Enzymes such as, sucrose synthase, phosphoglucomutase, transaldolase, enolase, pyruvate dehydrogenase, citrate synthase, succinyl‐coA ligase, fructose bis‐phosphate aldolase, phosphogluco kinase, phosphoglyceratemutase, fumarate dehydratase and malate dehydrogenase were up‐regulated up to 3.0‐fold during early and late stages following Foc inoculation in DV. However, they showed up to 2.0‐fold decrease in the roots of JG62. Other proteins from the same pathway such as fructokinase, succinate dehydrogenase and aconitase showed up to 2.56‐fold higher level in DV, while JG62 exhibited reduction by 1.4‐fold. Several proteins from the UPR pathway including Hsp70, luminal binding protein (BiP), calmodulin, component of SCF‐for SKP1‐Cullin‐F box protein and protein disulfide isomerase (PDI) increased up to 2.6‐fold in DV, while they showed 2‐fold reduction in JG62 upon Foc inoculation. The majority of these proteins were high in the early stage and low in the late stage in DV, while JG62 showed the opposite expression trend. Further, proteins induced by abiotic stress such as profilin and aquaporin PIP‐type 7a revealed 1.7 to 2.5‐fold increase in DV upon Foc inoculation compared with JG62. However, nuclear transcription factor‐Y (NF‐Y) had >30‐fold increase at both the stages in JG62 compared with DV.

Proteomic analyses further depicted that proteins involved in reactive oxygen species (ROS) generation such as ascorbate peroxidase, peroxiredoxin, dehydroascorbate reductase (DHAR), hydroxyacyl glutathione hydrolase, glutathione peroxidase, glutaredoxin, glutathione S‐transferase (GST), quinone oxidoreductase and copper amine oxidase (CuAO) showed 3.0 to 6.0‐fold increased expression in DV compared to JG62 exhibiting significant oxidative stress in chickpea after Foc inoculation. Additionally, enzymes involved in methionine metabolism, including methionine synthase, adenosine homocysteine hydrolase, adenosine kinase and AdoMet synthetase showed up to 1.6‐fold higher levels in the Foc‐inoculated DV at early and late stages, whereas JG62 displayed down‐regulation by >1.3‐fold at both the stages. In the present study, subsets of proteins participating in multiple branches of phenylpropanoid pathway such as lignin, flavonoid, isoflavonoid and phenolic biosynthesis were identified in Foc‐inoculated chickpea cultivars. Caffeic acid O‐methyltransferase (CAOMT) and caffeoyl‐CoA O‐methyltransferase (CCoAMT) showed up to 2.0 and 3.85‐fold increase, respectively, in DV compared to JG62. Similarly, enzymes from isoflavonoid biosynthesis such as chalcone synthase (CHS), chalcone isomerase (CHI), isoflavone synthase (IFS) and isoflavone reductase (IFR) revealed 1.8 to 5.0‐fold increase in DV than JG62.

Additionally, pathogenesis‐related (PR) proteins such as endo β‐1,3‐glucanase, major latex protein (MLP), major latex allergen hev b5 and Bet v1 showed up to 2.1‐fold higher expression in DV compared with JG62. Similarly, β‐gulcosidase, disease resistance response (DRR) protein‐206 and DRR‐49 had >5.0‐fold higher accumulation in DV than in JG62. Proteolytic chitinases offer antifungal properties and confer resistance to fungal pathogens. Chitinase, selenium binding protein (SBP) and glycine‐rich proteins revealed up to 1.8‐fold increased expression in DV compared with JG62. Interestingly, some PR proteins such as PR protein STH‐2 (2‐fold), thaumatin‐like protein PR‐5b (>5‐fold) and PR‐4A (>10‐fold) were very high at early stage in DV than JG62. However, these proteins exhibited reverse trend with 2.5 to 10‐fold increase in JG62 at late stages of Foc infection indicating their response to heavy wounding in chickpea. Some of the proteins involved in the stress signalling process such as ABA‐responsive protein, auxin‐binding protein ABP19a and Ran‐binding protein were increased by 2.1‐fold in DV compared with JG62. Additionally, 14‐3‐3 and H^+^‐ATPase showed up to 2.0‐fold higher levels in DV than in JG62, suggesting their role in defence response against Foc in chickpea roots.

### Metabolic profiling in chickpea root

**Figure 3:**
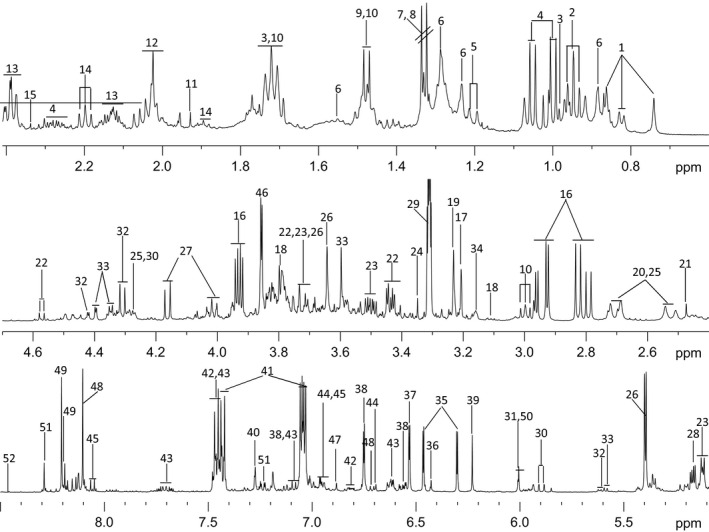
Typical NMR spectrum of chickpea root extract. Annotation with number and details of metabolites is provided in Table S2.

A typical annotated ^1^H‐NMR spectrum of chickpea root extract is depicted in Figure 3. The metabolite resonances were assigned according to the in‐house databases and previous publication (Fan, 1996). These were further confirmed with a series of 2D NMR spectra including ^1^H‐^1^H correlation spectroscopy (COSY), ^1^H‐^1^H total correlation spectroscopy (TOCSY), J‐resolved spectroscopy (JRES), ^1^H‐^13^C heteronuclear single quantum coherence spectroscopy (HSQC) and ^1^H‐^13^C heteronuclear multiple‐bond correlation (HMBC) with both ^1^H and ^13^C chemical shifts and signal multiplicities were as shown in Table S2. A total of 52 dominant metabolites were identified including amino acids (Ala, Val, Ile, Leu, Gln, Glu, Asn, Trp, Lys and GABA), sugars (glucose, sucrose, fructose, trehalose and salicin), organic acids (pyruvate, lactate, acetate, citrate, succinate, formate, fumarate, malate and guanidoacetate), nucleosides (adenosine, uridine, inosine, 5CMP and hypoxanthine) and phytoalexins (genistein and luteolin). The identification of few of these metabolites was confirmed by spiking with their known standards (Table S2).

### Differential metabolic alterations induced by Foc inoculation

**Figure 4:**
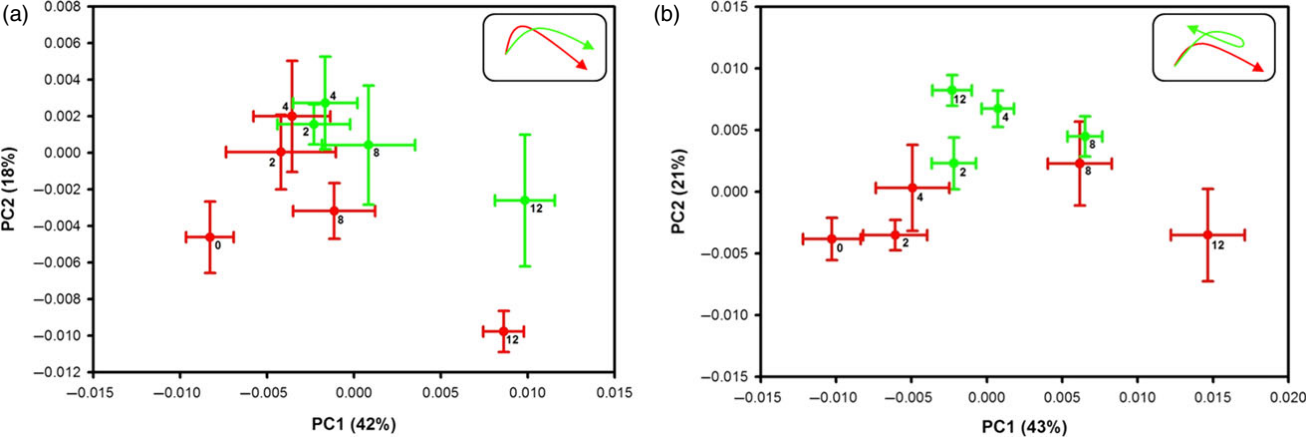
PCA trajectory plots. (a) resistant‐DV and (b) susceptible‐JG62 plants with their respective controls obtained from mean of PC1 and PC2 values at 2 to 12 DAI with error bars representing two standard deviations. Foc‐inoculated samples are in green while respective controls in red. Top right corner box indicates overall pattern.

**Figure 5:**
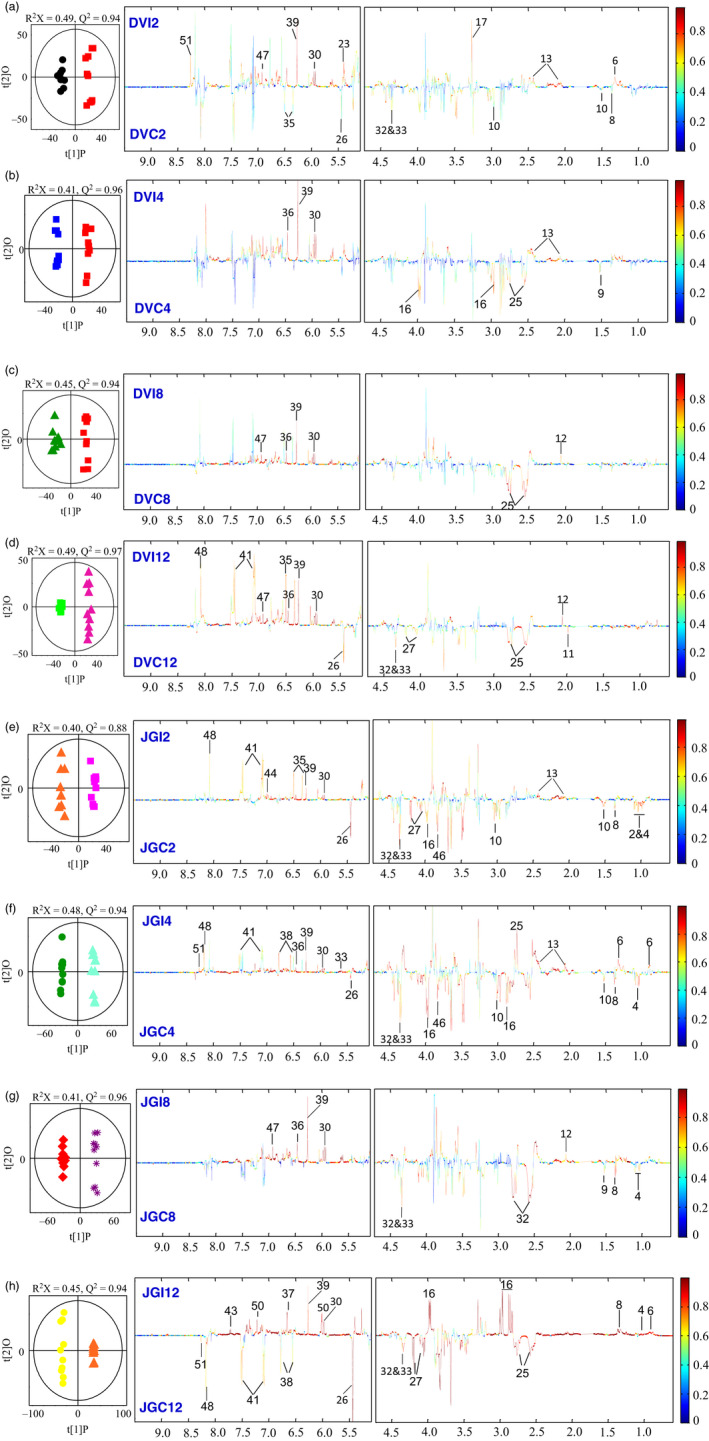
Pairwise comparison via OPLS‐DA. OPLS‐DA scores plots (left) and corresponding coefficient‐coded loadings plots (right) obtained from metabolic profiles of Foc‐inoculated (a–d) resistant‐DV and (e–h) susceptible‐JG62 cultivars and their respective controls at 2 to 12 DAI. The coloured scale in correlation coefficient (|r|: absolute values) plots shows the significance of metabolite variations discriminating between the Foc‐inoculated and control plants.

Principal component analysis (PCA) revealed significant metabolic changes between Foc‐inoculated resistant and susceptible cultivars at different time points. The averaged PCA scores were calculated for the first two PCs to construct the PCA trajectory that illustrated clear separation in metabolites from control and inoculated samples between two cultivars (Figure 4). Interestingly, the metabolic profiles obtained from the controls of both cultivars and the inoculated DV followed similar trajectory trends; however, the inoculated JG62 showed dramatic change in trajectory after 8 DAI (Figure 4). The metabolic changes resulted after Foc inoculations were further evaluated by constructing orthogonal projection to latent structures discriminant analysis (OPLS‐DA) models. The quality of models was indicated by the values of *R* ^2^ and *Q* ^2^ and cross‐validated with a CV‐ANOVA approach (*P *< 0.05) (Figures 5a and b) and permutation tests (Figure S2). All the significantly changed metabolites are annotated in the OPLS‐DA coefficient plots (Figures 5a and b) and summarized in Table S3.

Compared with the respective control, Foc inoculation induced marked reduction in the levels of amino acids including Thr, Ala, Lys and Asn at early stage in both the cultivars. However, their levels were significantly elevated in JG62 at late stage while DV remained unchanged (Figure 5). The decreased levels of Ile and Leu were observed at early stage and remained unchanged at late stage in JG62 after Foc inoculation. Interestingly, Val and Trp levels increased dramatically in the late stage of JG62 but remained unchanged in DV at both the stages (Figure 5). Similarly, two of the most important metabolites from nitrogen metabolism, such as Glu and Gln, were up‐regulated only at early stage of Foc inoculation in DV. However, JG62 revealed significantly higher accumulation of both of these amino acids at early and late stages. In addition, Foc inoculation resulted in decreased level of glucose in DV at early stage; however, no significant change was observed in both the cultivars at late stage. The levels of sucrose and fructose were decreased at early stage in JG62, followed by significant reduction at the later stage. It is of particular interest that the levels of some nucleotides including uridine and orotate were significantly decreased at the later stage of JG62 compared with DV. However, the opposite trend in the levels of adenosine and inosine was observed at the later stage of JG62. Compared with the controls, Foc inoculation induced significant reduction in the levels of malate and acetate in JG62 at the later stage. As for phytoalexins, genistein was increased in DV at the later stage but remained unchanged in JG62 and the level of luteolin decreased at the later stage of JG62 while DV was unaffected. A known antifungal compound, clotrimazole level increased in DV, but there was a marked reduction in JG62 at the later stage. In addition, Foc infection caused increase in quinone at early stage and phytosterol at the later stage of DV only.

### Comparative expression of candidate genes in root

**Figure 6:**
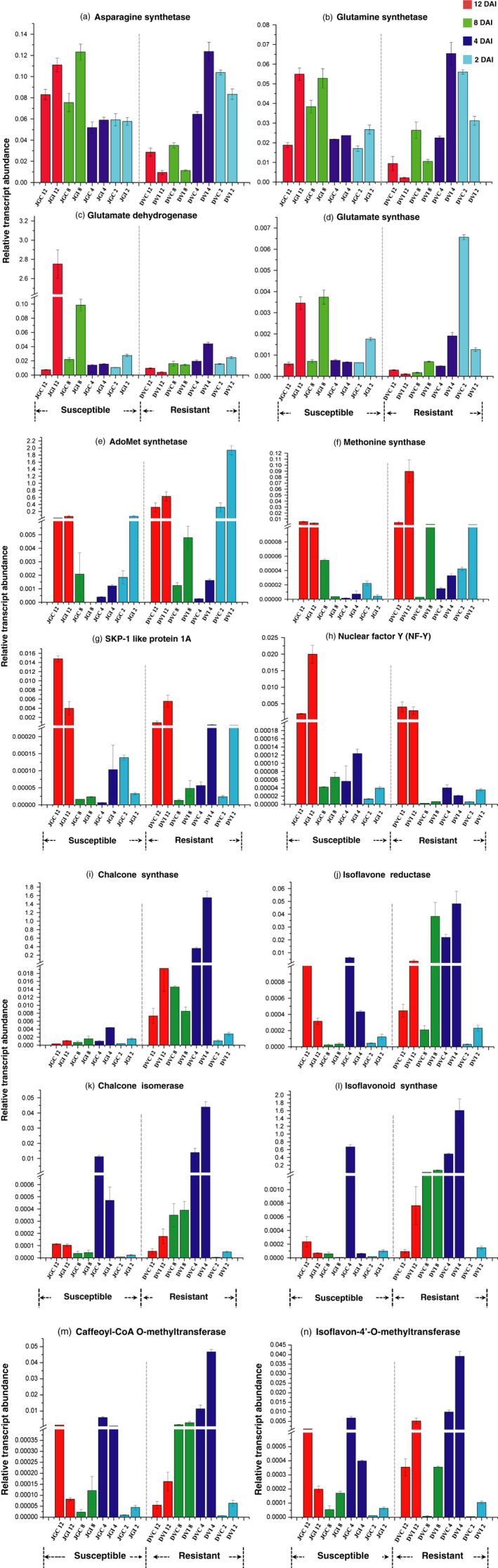
Quantitative real‐time PCR of various candidate genes. (a–n) expression variation in each gene observed from chickpea root of Foc‐inoculated resistant‐DV and susceptible‐JG62 cultivars as compared to their respective controls at 2 to 12 DAI.

To obtain complementary information of transcriptional variations, we examined expression levels of key genes (Figure 6) involved in various metabolic pathways based on our proteomic and metabolomic findings. These included genes from nitrogen mobilization (glutamate dehydrogenase‐GDH, glutamate synthase, glutamine synthase and asparagine synthase), stress response (NF‐Y and SKP1‐like protein 1A), methionine metabolism (methionine synthase and AdoMet synthetase), lignin and phytoalexin biosynthetic pathways (CCoAMT, CHS, CHI, isoflavone 4′‐O‐methyltransferase, IFS and IFR). *GDH* expression markedly increased by >200 fold in the inoculated JG62 at the later stage. Similarly, significant up‐regulation (up to 4‐fold) of glutamine synthetase, asparagine synthetase and glutamate synthase was observed in the Foc‐inoculated JG62 (Figure 6a–d). However, candidate genes from methionine metabolism such as Adomet synthetase and methionine synthase revealed >3 and 10‐fold enhanced expression, respectively, in DV (Figure 6e and f). Also, important genes from lignin and phytoalexin biosynthetic pathway displayed higher expression in DV compared with JG62 (Figures 6i–n). On the contrary, abiotic stress induced *NF‐Y* gene showed >8‐fold higher expression in JG62 (Figure 6h), while *SKP*1 was expressed more in DV (Figure 6g).

### Intense lignification is associated with Foc resistant phenotype

After Foc inoculation, differential lignin deposition in the root tissue of DV and JG62 was observed as the time progressed, wherein DV exhibited intense lignification compared with JG62 (Figure S3). The fungal pathogen invaded the susceptible cultivar and blocked the vascular tissue completely by 12 DAI, leading to wilting of JG62 plants.

## Discussion

### Foc induced remodelling in energy metabolism and nitrogen mobilization

Obligate biotrophs depend on host metabolism for nutrient uptake, which in turn determine their pathogenicity within the host. In the present study, proteins involved in glycolysis and TCA cycle were up‐regulated in DV while down‐regulated in JG62. We also observed steep alteration in primary metabolites (amino acids and sugars) specifically in JG62. Sugars affect disease susceptibility often favouring disease development while playing a critical role in innate defence pathways involving metabolic regulation (Bolouri Moghaddam and Van den Ende, 2012). In the present study, rapid decrease in sugars such as sucrose and fructose was observed upon Foc infection in both the cultivars; however, this was more predominant in the JG62. Similar rapid reduction in the levels of these sugars was also reported in sunflower upon infection with *Botrytis cinerea* (Dulermo *et al*., 2009b). These results emphasized the regulatory role of sugars in the metabolic reprogramming and subsequently higher expression of proteins from glycolysis and TCA cycle endowed DV plants to successfully combat Foc invasion. Moreover, supportive evidence could be derived from earlier reports of *F. oxysporum* induced up‐regulation of various ESTs from sugar metabolism in chickpea (Ashraf *et al*., 2009; Gupta *et al*., 2010, 2013a).

Nitrogen plays an essential role in the nutrient relationship between plants and pathogens. Modulation in amino acid concentration due to nitrogen mobilization after pathogen infection to plant has been reported (Dulermo *et al*., 2009a; Tavernier *et al*., 2007). Proteomics studies on wheat–*F. graminearum* exhibited increase expression in proteins from amino acid, carbon and nitrogen metabolism (Wang *et al*., 2005; Zhou *et al*., 2006). Such alteration with significantly low amino acid content was also observed in tomato and sunflower after infection of *B. cinerea* and *Sclerotinia sclerotiorum*, respectively (Berger *et al*., 2004; Jobic *et al*., 2007). Current study showed significant decrease in the concentration of various amino acids in the susceptible cultivar, which suggested that the fungus probably utilized them for its establishment and proliferation inside the host. However, probably due to Foc sporulation, levels of amino acids were increased at the later stage in JG62. Hence, the role of nitrogen mobilization was further scrutinized in chickpea–Foc interaction by gene‐expression analysis of four representative enzymes from nitrogen mobilization *viz*. glutamate synthase, glutamate dehydrogenase, glutamine synthetase and asparagine synthetase. Significant up‐regulation of these genes in JG62 compared to DV correlated well with the metabolomics outcome. Consistently, proteomic analysis also revealed higher level of sucrose synthase and glutamine synthase following Foc inoculation. Thus, proteomic, metabolomic and gene‐expression results together indicated that the process of nitrogen mobilization could be critical in the establishment of Foc infection in the susceptible plants.

### Stress responsive proteins in chickpea root

During stress, accumulation of unfolded proteins increases in endoplasmic reticulum (ER) and results in triggering the unfolded protein response (UPR) to remove the misfolded proteins by ubiquitin–proteasome pathway. Thus, UPR not only helps to avert the cytotoxic impact of misfolded proteins, but also assists to relieve stress and reinstate normal functions in ER (Ye *et al*., 2011). Up‐regulation of critical proteins from UPR pathway such as Hsp70, BiP, calmodulin, SKP1 and PDI in DV could suggest their coordinate response. In *Arabidopsis thaliana* roots, swelling of ER and vacuolar collapse resulting in ER stress and cell death were observed during fungal colonization (Qiang *et al*., 2012). Similarly, PDI level was higher in wheat plants upon *Puccinia striiformis* inoculation (Maytalman *et al*., 2013). This study also reinforced that during defence response, there was constant requirement for proteins stabilization in the process of folding, assembly, vesicle trafficking and secretion. Collectively, these outcomes suggested the importance of an efficient UPR pathway utilization in the resistant chickpea against Foc.

During stress conditions, aquaporins such as PIP are involved in water transport in plant. Current investigation revealed significant increase in PIP‐7a level at both the stages in DV, while drastic reduction was observed in JG62. This could have resulted in better water conductance in DV and helped the resistant plant to overcome fungal attack. On the contrary, diminished water transport with reduced aquaporin in xylem due to fungal spread in the susceptible plant could have eventually turned into wilting. Furthermore under water‐limited conditions, nuclear factor‐Y (NF‐Y) family and ABA‐responsive protein have shown to be up‐regulated in *Arabidopsis* (Nelson *et al*., 2007). We observed high expression of these proteins at both the stages in JG62 compared with DV. Thus, present findings indicated constrain in water uptake in JG62 due to clogged xylem after fungal invasion that potentially increased the susceptibility to Foc.

### Early recognition of Foc leads to ROS generation and lignosuberization

Generation of ROS is one of the earliest cellular responses to pathogen recognition and/or infection (Gupta *et al*., 2013b). The enzymes involved in ROS production such as peroxidase, DHAR, hydroxyacyl glutathione hydrolase, glutathione peroxidase, glutaredoxin, GST, quinone oxidoreductase and CuAO were significantly increased in DV compared with JG62 in the current investigation. It has been demonstrated that CuAO and peroxidases functionally correlate in lignosuberization process (Angelini *et al*., 1993; Scalet *et al*., 1991) and the inhibitors of CuAO result in decreased defence response (Rea *et al*., 1998, 2002). Additionally, Raju *et al*. (2008) found more lignification in the resistant chickpea cultivar compared with the susceptible one upon Foc infection. In the present study also, intense lignin deposition on the root cortex of Foc‐inoculated resistant cultivar was observed (Figure S3). Taken together, ROS generation and higher expression of CuAO suggested that Foc triggered hydrogen peroxide generation and lignosuberization process leading to initiation of defence response in DV. Secondly, monolignol biosynthesis also plays critical role in the host defence mechanism through lignification, making cell wall more resistant to the pathogen penetration. Reduction in the monolignol biosynthesis through co‐silencing of *CAOMT* and *CCoAMT* in wheat led to the higher penetration by *Blumeria graminis* (Bhuiyan *et al*., 2009). We also found up‐regulation of these two lignin biosynthetic enzymes in DV than JG62 suggesting increased lignin deposition that might provide resistance against Foc.

### Crucial role of methionine metabolism in Foc resistance

In the Yang cycle, methionine synthase converts homocysteine to methionine contributing in synthesis of Adomet by AdoMet synthetase. AdoMet can also lead to ethylene by 1‐aminocyclopropane‐1‐carboxylic acid (ACC) synthase and ACC oxidase. However, down‐regulation of ACC oxidase by 1.5‐fold in both the cultivars indicated that preferential AdoMet pool was not channelized towards ethylene production. A recent proteomic study has shown that increased expression of AdoMet synthetase and lower level of ACC oxidase resulted in higher methionine recycling and lower ethylene biosynthesis in rice roots against *Herbaspirillum seropedicae* (Alberton *et al*., 2013). Silencing of AdoHcy hydrolase in transgenic tobacco plants confirmed its role in defence mechanism against pathogens (Masuta *et al*., 1995). Similarly, Kawalleck *et al*. (1992) identified mRNAs for AdoMet synthetase and AdoHcy hydrolase in parsley plant upon fungal infection. Altogether, current study indicated close association between pathogen defence and increased level of activated methyl groups during chickpea–Foc interplay.

Further, highly methylesterified pectin is required for normal plant cell wall, while it is de‐esterified by pectin methyl esterase (PME) which leads to increased vulnerability of plant cell wall to the pathogen invasion (Lionetti *et al*., 2007). Our proteomics results showed increased expression of PME at both the stages in JG62 compared with DV. Earlier studies with either silencing of PME or overexpression of PME inhibitors in plants demonstrated negative role of PME in the pathogen resistance (An *et al*., 2008; Ma *et al*., 2013). Likewise, plant sterols are structurally related to cholesterol and control mechanical property of cell membrane (Hodzic *et al*., 2008) and also serve as substrate for several metabolic pathways (Itkin *et al*., 2013). Interestingly, our metabolomic data also revealed high phytosterol at later stages in DV. However, there was no significant change in JG62 except at 8 DAI. The stability of the plant cell wall is also maintained by actin binding cytoskeleton protein such as Profilin, which showed higher expression in DV compared with JG62 at late stage. Overall, altered methionine metabolism affected methyl esterification of pectin in Foc‐infected susceptible roots, while stronger plant cell wall with normal pectin and cytoskeleton proteins could have helped the resistant chickpea cultivar with better defence against Foc.

### Foc resistance is mediated by phenylpropanoid pathway

Plants respond to pathogen challenge by increased activation of the phenylpropanoid pathway leading to flavanoids, isoflavonoids and phenolics biosynthesis. They play multiple roles in plant–pathogen interaction including precursors for the defence‐related phytoalexins and signal molecules in response to pathogen infection. As detailed in the results, enzymes involved in this pathway such as CHS, CHI, IFR and IFS were up‐regulated in DV as compared to JG62. In the transgenic soybean roots, RNAi silencing of *CHS* gene showed decrease in total isoflavonoids as well as reduced resistance to fungal pathogens (Lozovaya *et al*., 2006; Subramanian *et al*., 2005). Additionally, Naoumkina *et al*. (2007) and Farag *et al*. (2008) showed that fungal extract induced higher levels of *CHI*, *IFS* and *IFR* leading to the production of phytoalexin in Medicago cell suspension culture to combat the pathogen. Consistently, the accumulation of genistein, luteolin and quinone identified by metabolome analysis in the present study correlated well with the proteomic data. Thus, the accumulation of isoflavonoid biosynthetic proteins and metabolites in DV suggested their potential involvement in Foc resistance.

### Modulation of defence‐related proteins and metabolites upon Foc inoculation

In the present study, we observed quantitative variation in many defence‐related proteins such as endo β‐1,3‐glucanase, MLP, hev b5 and Bet v1, β‐gulcosidase, DRR‐206, DRR‐49, chtinases, SBP, PR10, STH‐2, PR4a, PR‐5b, 14‐3‐3 and H^+^‐ATPase in Foc‐inoculated chickpea cultivars. Many previous studies in other plant–pathogen interactions have also demonstrated the importance of such proteins in plant defence. Lytle *et al*. (2009) and Gurjar *et al*. (2012) have reported that Bet v1 protein is responsible for resistance towards pathogen. Similarly, β‐1,3‐glucanase is involved in plant defence response against pathogen (Shetty *et al*., 2009; Ward *et al*., 1991). Previously two independent studies have shown that β‐glucosidase, DRR‐206 and DRR‐49 proteins contribute to lignification process (Burlat *et al*., 2001; Hosel and Barz, 1975). Increased chitinase activity was also reported earlier after Foc inoculation in the resistant chickpea cultivar (Giri *et al*., 1998). Similarly, overexpressed SBP in rice provided more resistance against rice blast fungus (Sawada *et al*., 2004). Another important defence‐related protein, 14‐3‐3, is known to be associated with hypersensitive cell death in pathogen incompatible cultivars (Roberts, 2003). In our earlier study, up‐regulation of 14‐3‐3 transcripts was detected in Foc‐inoculated resistant chickpea roots (Nimbalkar *et al*., 2006). Thus, up‐regulation of 14‐3‐3 and H^+^‐ATPase suggested their roles in activating hypersensitivity response in chickpea leading to Foc resistance. Apart from the above‐mentioned proteins, our metabolite analysis revealed increase level of clotrimazole in Foc‐inoculated DV at late stage compared to JG62. This is an interesting observation, clotrimazole being an anthropogenic antifungal compound and needs further experimentation. Thus, the pathogen attack triggered expression of defence‐related genes, secondary metabolites with antimicrobial nature and PR proteins in the chickpea–Foc interactions.

In summary, our integrated approach of label‐free quantitative proteomics, ^1^H‐NMR metabolomics and candidate gene‐expression analysis has facilitated to understand the defence mechanism in chickpea against Foc. Most of the proteins/metabolites from various metabolic processes such as energy metabolism, isoflavonoid/flavonoid biosynthesis pathway and lignin biosynthesis that lead to defence were up‐regulated in the resistant plant compared with the susceptible one. Further, elevated levels of some of the proteins from the UPR pathway during incompatible interaction indicated that proper protein folding and transport might be crucial for the plant's survival. Some of the proteins/metabolites such as isoflavone reductase and isoflavone synthase leading increased phytoalexins levels; CAOMT and CCoMT involved in lignosuberization and increased methionine synthase for efficient methylation process in the resistant plant could have helped against the pathogen. Overall, above findings improve our understanding on the metabolic reprogramming during the wilt disease progression.

## Experimental procedures

### Plant material

Seeds of wilt resistant (Digvijay, DV) and susceptible (JG62, JG) chickpea cultivars as well as pathogenic culture of Foc race 1 were obtained from Mahatma Phule Krishi Vidyapeeth (MPKV), Rahuri, India. All the steps of plant growth and pathogen inoculation were followed as previously described (Kumar *et al*., 2015) and divided into two groups, control or mock (sterile water) inoculated and Foc (~10^6^ spores/mL) inoculated. The workflow for sample collection/data analysis is shown in Figure S4. As the pathogen is reported to colonize the xylem vessels 2 days postinoculation (Gupta *et al*., 2010), the root tissues were collected at 2, 4, 8 and 12 DAI. Whole roots of chickpea was used in equal amount from the resistant and susceptible cultivars at specific time points simultaneously for the proteomics and metabolomics analysis; because the fungal hyphae might not be localized but distributed in the whole root xylem tissue (Jimenez‐Fernandez *et al*., 2013). In each group, a pool of 10 plants comprised one biological replicate. For proteomics analyses, tissues from three biological replicates were analysed, while ten biological replicates were analysed for metabolomics analysis. The harvested tissues were stored at −80 °C till further use.

### Protein extraction and mass spectrometry analysis

Total proteome of root tissue was extracted as described by Isaacson *et al*. (2006). Protein pellets were solubilized in 50 mm ammonium bicarbonate buffer containing 0.1% Rapigest (Waters, Milford, MA). The dissolved proteins were reduced and alkylated by DTT and iodoacetamide, respectively, followed by overnight tryptic hydrolysis at 37 °C using Promega sequencing grade trypsin. The digested peptides were analysed with LC‐MS^E^ workflow using nano‐ACQUITY online coupled to a SYNAPT HDMS system (Waters). Nano‐LC separation was performed with symmetry C18 trapping column (180 μm × 20 mm, 5 μm) and bridged‐ethyl hybrid (BEH) C18 analytical column (75 μm × 250 mm, 1.7 μm). The binary solvent system comprised solvent A (0.1% formic acid in water), and solvent B (0.1% formic acid in acetonitrile). Each sample (500 ng) was initially applied to the trapping column and desalted by flushing with 1% solvent B for 1 min at a flow rate of 15 μL/min. Elution of the tryptic digested sample was performed at a flow rate of 300 nL/min by increasing the solvent B concentration from 3% to 40% over 90 min. Before data acquisition, the mass analyser was calibrated using Glu‐fibrinopeptide B (Sigma‐Aldrich, Steinheim, Germany) from m/z 50 to 1990. The Glu‐fibrinopeptide B (GFP‐B) was delivered at 500 fmole/μL to the mass spectrometer via a NanoLockSpray interface using the auxiliary pump of the nano‐ACQUITY system at every 30 s interval for lock mass correction during data acquisition. Data‐independent acquisition was performed (LC‐MS^E^) as described by Patel *et al*. (2009).

As accuracy and reproducibility in mass measurement are critical in data acquisition during large‐scale proteomic experiments, PCA was used to assess the quality of the measurement in terms of replicate similarity. The replicates of each sample were clustered together reflecting inherent similarities between samples (Figure S5a). In addition, linear response and reproducibility of measurement of the quantitative proteomic data acquisition were tested by plotting two replicates (Figure S5b), whereas Figure S5c showed that data have been acquired below 3 ppm mass accuracy. Further, the percent coefficient of variance of retention time (% CV‐RT) was calculated to assess the separation stability and coefficient of variance of 0.3 min, which also suggested stability in chromatographic separation (Figure S5d).

### Analysis of quantitative proteomics data

The acquired LC‐MS^E^ data were processed using the ProgenesisQI for Proteomics software (Waters). Protein identifications were obtained by searching the genomic databases of chickpea (http://www.icrisat.org/) and *Fusarium oxysporum* (http://www.broadinstitute.org/). LC‐MS^E^ data were searched with a fixed carbamidomethyl modification for cysteine residues, along with a variable modification for oxidation of methionine, N‐terminal acetylation, deamination of asparagine and glutamine and phosphorylation of serine, threonine and tyrosine. The ion accounting search algorithm within ProgenesisQI for Proteomics software was used which has been developed specifically for searching data‐independent MS^E^ data sets and described by Li *et al*. (2009). The ion accounting search parameters were (a) precursor and product ion tolerance: automatic setting, (b) minimum number of product ion matches per peptide: 3, (c) minimum number of product ion matches per protein: 7, (d) minimum number of peptide matches per protein: 1 and (e) missed tryptic cleavage sites: 1. False‐positive rate was set at 1%. Search results of the proteins and the individual MS/MS spectra with a confidence level at or >95% were accepted. Label‐free quantitation of identified protein was done on the basis of spiked bovine serum albumin (BSA) protein.

### Clustering of identified proteins

Data were normalized by spiked BSA (50 fmoles), and relative accumulation differences were determined for proteins having differential expression. Sum of three replicates of inoculated samples was divided by that of the respective controls. This established a ratio of accumulation of a protein in plants upon Foc inoculation compared with mock‐inoculated control plants. The log_2_‐transformed ratio (susceptible/control and resistant/control) pairs were clustered by the application of SplineCluster (Heard *et al*., 2006), a Bayesian model‐based hierarchical clustering algorithm for time series data.

### Gene ontology enrichment analysis

Protein functional annotation was determined using Blast2GO (Conesa *et al*., 2005) and for each cluster, GO enrichment analysis was carried out using BiNGO 2.3 plugin tool in Cytoscape version 2.8 (Maere *et al*., 2005). Over‐represented GO biological process categories were identified using a hypergeometric test with a significance threshold of 0.05 after Benjamini and Hochberg false discovery rate correction (Benjamini and Hochberg, 1995) using the annotated chickpea genome as the reference set.

### Metabolite extraction and NMR measurement

Plant root tissue was ground well in liquid nitrogen by using bead beater (Retsch GmbH, Retsch‐Allee, Germany) and lyophilized. The powdered root tissues (~50 mg) were extracted with 0.75 mL of CD_3_OD and 0.75 mL of 10 mm KH_2_PO_4_ buffer (pH 6.0) containing sodium3‐trimethlysilyl [2,2,3,3‐D_4_] propionate (TSP) as described previously (Kim *et al*., 2010). After ultrasonication for 20 min and centrifugation at 12 000 g for 10 min at room temperature (~25 °C), 0.5 mL of supernatant was collected for NMR detection. ^1^H NMR spectra of root extracts were acquired at 25 °C on a Bruker AV II 500 spectrometer (Bruker Biospin, Rheinstetten, Germany) operating at 500.13 MHz for ^1^H. A standard water‐suppressed one‐dimensional NMR spectrum was recorded using *noesypr1d* pulse sequence (RD‐90°‐*t*_1_‐90°‐*t*m‐90°‐acquisition) with the recycle delay of 6 s and the mixing time (*t*_m_), of 50 ms. Typically, 90° pulse was set to about 15 μs and 256 transients were collected into 48K data points for each spectrum with a spectral width of 16 ppm. All spectra were referenced to chemical shift of TSP (δ = 0.00). For the metabolite assignment purpose, a range of two‐dimensional NMR spectra were recorded for selected samples including COSY, TOCSY, HSQC and HMBC. In COSY and TOCSY experiments, respective 64 and 32 transients were collected into 2K data points for each of 256 increments with the spectral width of 2426 Hz for both dimensions. Magnitude mode was used with gradient selection for the COSY experiments, whereas the *mlevgpphw5* pulse program was employed as the spin‐lock scheme in the phase sensitive mode, with the mixing time of 60 ms, for TOCSY. Both HSQC and HMBC spectra were acquired using the gradient‐selected sequences. In HSQC experiment, 80 transients were collected into 1K data points for each of 140 increments. In HMBC experiment, 160 transients were collected into 2K data points for each of 256 increments. The spectral widths were 2426 Hz for ^1^H and 9809 Hz for ^13^C in HSQC and HMBC experiments. Confirmation of resonance assignments were performed for few metabolites by spiking the samples with known standards (Table S2).

### NMR spectra processing and multivariate data analysis

All the ^1^H NMR spectra were manually corrected for phase and baseline distortions using TOPSPIN (v2.1; Bruker Biospin) and calibrated for chemical shift drifting by in‐house‐developed script for MATLAB (The Mathworks, Natick, MA). The spectral region δ 0.5–9.5 was divided into bins with width of 0.002 ppm (1.0 Hz) using AMIX software (v3.8.3; Bruker Biospin GmbH, Germany). The region δ 4.727–5.089 ppm was discarded to remove the effects of imperfect water presaturation. The areas of the remaining bins were normalized to total sum of intensity for each spectrum to compensate for the overall concentration differences prior to statistical data analysis. Multivariate data analyses were carried out with SIMCA‐P+ v 12.0 software package (Umetrics, Umeå, Sweden). PCA was performed on the mean‐centred NMR data to inspect overall data distributions and possible outliers. Using the NMR data as the X‐matrix and group information as Y‐matrix, OPLS‐DA was carried out with unit variance scaling (Trygg, 2002; Xiao *et al*., 2008). The OPLS‐DA models were 7‐fold cross‐validated and the quality of the model was described by the parameters R^2^X, representing the total explained metabolic variables and Q^2^, indicating the model predictability. The models were further evaluated with a CV‐ANOVA approach (*P *< 0.05) and permutation tests. To facilitate interpretation of the results, back‐transformation (Cloarec *et al*., 2005) of the loadings generated from the OPLS‐DA was performed prior to generating the loadings plots, which were colour‐coded with the Pearson linear correlation coefficients of variables (or metabolites) using an in‐house‐developed script for MATLAB (The Mathworks) (Wang *et al*., 2007). The colour‐coded correlation coefficient indicates the significance of the metabolite contribution to the class separation, with hot colours (e.g. red) being more significance of the metabolite contributions to the group classification than cold ones (e.g. blue). In this study, a correlation coefficient cut‐off value of 0.602 (i.e., *N *= 10, |*r*| > 0.602) was used for the statistical significance based on the discrimination significance at the level of *P *< 0.05, which was determined according to the discriminating significance of the Pearson's product–moment correlation coefficient (Cloarec *et al*., 2005).

### Quantitative real‐time PCR analysis

Total RNA was extracted from 100 mg root tissue by using TRI Reagent (Sigma‐Aldrich). First strand cDNA synthesis was performed using the High Capacity cDNA Reverse Transcription Kits (Applied Biosystems, Foster City, CA) with 3 μg of DNase1 treated total RNA using oligo (dT) primer following manufacturer's protocol. Gene‐specific primers were designed using Primer Express (v2.0) software (Applied Biosystems) and listed in Table S4. Real‐time PCR was carried out as earlier described (Barvkar *et al*., 2012) using FastStart universal SYBR green master mix (Roche, Mannheim, Germany) with 7900HT Fast real‐time PCR system (Applied Biosystems). The Initiation factor 4α (*IF4*α) gene was used as internal standard or reference gene (Garg *et al*., 2010).

### Lignin staining

Lignin accumulation within root tissue after Foc inoculation was detected using the phloroglucinol/hydrochloric acid stain as described by MauchMani and Slusarenko (1996). The control and Foc‐inoculated chickpea roots were subjected to transverse sections with a scalpel and immersed in 1 mL of 1% phloroglucinol in 6N HCl for 5 min, and the lignin staining was visualized under light microscope.

## Supporting information


**Figure S1** The Foc resistant‐DV and susceptible‐JG62 (JG) chickpea plants at early and late stages after inoculation.

**Figure S2** Permutation test results for OPLS‐DA models with two components and 200 permutations.

**Figure S3** Pattern of lignification in cross sections of chickpea root tissue from resistant‐DV and susceptible‐JG62 at different Foc inoculation stages using phloroglucinol/hydrochloric acid stain.

**Figure S4** Design of experiment with details of sample preparation, tissue collection stages, approaches and data analysis.

**Figure S5** Protein quality control measurements.

**Table S1** (a) List of the identified proteins using LC‐MSE. (b) List of 481 differentially accumulated proteins identified using LC‐MSE. (c) List of 481 differentially expressed proteins with their actual fold changes.

**Table S2** Assignments of metabolites from ^1^H‐NMR analysis.

**Table S3** Metabolites with significant contribution to the discriminations between inoculated and control plants based on OPLS‐DA.

**Table S4** List of primers used in quantitative real‐time PCR.

Supplementary Legends

## References

[pbi12522-bib-0001] Alberton, D. , Muller‐Santos, M. , Brusamarello‐Santos, L.C.C. , Valdameri, G. , Cordeiro, F.A. , Yates, M.G. , Pedrosa, F.D. *et al*. (2013) Comparative proteomics analysis of the rice roots colonized by *Herbaspirillum seropedicae* strain SmR1 reveals induction of the methionine recycling in the plant host. J. Proteome Res. 12, 4757–4768. 23971515 10.1021/pr400425f

[pbi12522-bib-0002] An, S.H. , Sohn, K.H. , Choi, H.W. , Hwang, I.S. , Lee, S.C. and Hwang, B.K. (2008) Pepper pectin methylesterase inhibitor protein CaPMEI1 is required for antifungal activity, basal disease resistance and abiotic stress tolerance. Planta, 228, 61–78. 18327607 10.1007/s00425-008-0719-z PMC2413075

[pbi12522-bib-0003] Angelini, R. , Bragaloni, M. , Federico, R. , Infantino, A. and Portapuglia, A. (1993) Involvement of polyamines, diamine oxidase and peroxidase in resistance of chickpea to *Ascochyta rabiei* . J. Plant Physiol. 142, 704–709.

[pbi12522-bib-0004] Anjaiah, V. , Cornelis, P. and Koedam, N. (2003) Effect of genotype and root colonization in biological control of Fusarium wilts in pigeonpea and chickpea by *Pseudomonas aeruginosa* PNA1. Can. J. Microbiol. 49, 85–91. 12718396 10.1139/w03-011

[pbi12522-bib-0005] Ashraf, N. , Ghai, D. , Barman, P. , Basu, S. , Gangisetty, N. , Mandal, M.K. , Chakraborty, N. *et al*. (2009) Comparative analyses of genotype dependent expressed sequence tags and stress‐responsive transcriptome of chickpea wilt illustrate predicted and unexpected genes and novel regulators of plant immunity. BMC Genom. 10, 415. 10.1186/1471-2164-10-415 PMC2755012 19732460

[pbi12522-bib-0006] Barvkar, V.T. , Pardeshi, V.C. , Kale, S.M. , Kadoo, N.Y. , Giri, A.P. and Gupta, V.S. (2012) Proteome profiling of flax (*Linum usitatissimum*) seed: characterization of functional metabolic pathways operating during seed development. J. Proteome Res. 11, 6264–6276. 23153172 10.1021/pr300984r

[pbi12522-bib-0007] Benjamini, Y. and Hochberg, Y. (1995) Controlling the false discovery rate ‐ a practical and powerful approach to multiple testing. J. R. Stat. Soc. Series B Stat. Methodol 57, 289–300.

[pbi12522-bib-0008] Berger, S. , Papadopoulos, M. , Schreiber, U. , Kaiser, W. and Roitsch, T. (2004) Complex regulation of gene expression, photosynthesis and sugar levels by pathogen infection in tomato. Physiol. Plant. 122, 419–428.

[pbi12522-bib-0009] Bhuiyan, N.H. , Selvaraj, G. , Wei, Y.D. and King, J. (2009) Gene expression profiling and silencing reveal that monolignol biosynthesis plays a critical role in penetration defense in wheat against powdery mildew invasion. J. Exp. Bot. 60, 509–521. 19039100 10.1093/jxb/ern290 PMC2651457

[pbi12522-bib-0010] Bollina, V. , Kushalappa, A.C. , Choo, T.M. , Dion, Y. and Rioux, S. (2011) Identification of metabolites related to mechanisms of resistance in barley against *Fusarium graminearum*, based on mass spectrometry. Plant Mol. Biol. 77, 355–370. 21830145 10.1007/s11103-011-9815-8

[pbi12522-bib-0011] Bolouri Moghaddam, M.R. and Van den Ende, W. (2012) Sugars and plant innate immunity. J. Exp. Bot. 63, 3989–3998. 22553288 10.1093/jxb/ers129

[pbi12522-bib-0012] Burlat, V. , Kwon, M. , Davin, L.B. and Lewis, N.G. (2001) Dirigent proteins and dirigent sites in lignifying tissues. Phytochemistry, 57, 883–897. 11423139 10.1016/s0031-9422(01)00117-0

[pbi12522-bib-0013] Chen, F.F. , Zhang, J.T. , Song, X.S. , Yang, J. , Li, H.P. , Tang, H.R. and Liao, Y.C. (2011) Combined metabonomic and quantitative real‐time PCR analyses reveal systems metabolic changes of *Fusarium graminearum* induced by Tri5 gene deletion. J. Proteome Res. 10, 2273–2285. 21413710 10.1021/pr101095t

[pbi12522-bib-0014] Cho, S.H. , Lee, J. , Jung, K.H. , Lee, Y.W. , Park, J.C. and Paek, N.C. (2012) Genome‐wide analysis of genes induced by *Fusarium graminearum* infection in resistant and susceptible wheat cultivars. J. Plant Biol. 55, 64–72.

[pbi12522-bib-0015] Cloarec, O. , Dumas, M.E. , Trygg, J. , Craig, A. , Barton, R.H. , Lindon, J.C. , Nicholson, J.K. *et al*. (2005) Evaluation of the orthogonal projection on latent structure model limitations caused by chemical shift variability and improved visualization of biomarker changes in H^1^ NMR spectroscopic metabonomic studies. Anal. Chem. 77, 517–526. 15649048 10.1021/ac048803i

[pbi12522-bib-0016] Conesa, A. , Gotz, S. , Garcia‐Gomez, J.M. , Terol, J. , Talon, M. and Robles, M. (2005) Blast2GO: a universal tool for annotation, visualization and analysis in functional genomics research. Bioinformatics, 21, 3674–3676. 16081474 10.1093/bioinformatics/bti610

[pbi12522-bib-0017] Dulermo, T. , Bligny, R. , Gout, E. and Cotton, P. (2009a) Amino acid changes during sunflower infection by the necrotrophic fungus *B. cinerea* . Plant Signal. Behav. 4, 859–861. 19847103 10.4161/psb.4.9.9397 PMC2802803

[pbi12522-bib-0018] Dulermo, T. , Rascle, C. , Chinnici, G. , Gout, E. , Bligny, R. and Cotton, P. (2009b) Dynamic carbon transfer during pathogenesis of sunflower by the necrotrophic fungus *Botrytis cinerea*: from plant hexoses to mannitol. New Phytol. 183, 1149–1162. 19500266 10.1111/j.1469-8137.2009.02890.x

[pbi12522-bib-0019] Fan, W.M.T. (1996) Metabolite profiling by one‐ and two‐dimensional NMR analysis of complex mixtures. Prog. Nucl. Magn. Reson. Spectrosc. 28, 161–219.

[pbi12522-bib-0020] Farag, M.A. , Huhman, D.V. , Dixon, R.A. and Sumner, L.W. (2008) Metabolomics reveals novel pathways and differential mechanistic and elicitor‐specific responses in phenylpropanoid and isoflavonoid biosynthesis in *Medicago truncatula* cell cultures. Plant Physiol. 146, 387–402. 18055588 10.1104/pp.107.108431 PMC2245840

[pbi12522-bib-0021] Garg, R. , Sahoo, A. , Tyagi, A.K. and Jain, M. (2010) Validation of internal control genes for quantitative gene expression studies in chickpea (*Cicer arietinum* L.). Biochem. Bioph. Res. Commun. 396, 283–288. 10.1016/j.bbrc.2010.04.079 20399753

[pbi12522-bib-0022] Giri, A.P. , Harsulkar, A.M. , Patankar, A.G. , Gupta, V.S. , Sainani, M.N. , Deshpande, V.V. and Ranjekar, P.K. (1998) Association of induction of protease and chitinase in chickpea roots with resistance to *Fusarium oxysporum* f.sp. *ciceri* . Plant. Pathol. 47, 693–699.

[pbi12522-bib-0023] Golkari, S. , Gilbert, J. , Prashar, S. and Procunier, J.D. (2007) Microarray analysis of *Fusarium graminearum*‐induced wheat genes: identification of organ‐specific and differentially expressed genes. Plant Biotechnol. J. 5, 38–49. 17207255 10.1111/j.1467-7652.2006.00213.x

[pbi12522-bib-0024] Gowda, S.J.M. , Radhika, P. , Kadoo, N.Y. , Mhase, L.B. and Gupta, V.S. (2009) Molecular mapping of wilt resistance genes in chickpea. Mol. Breeding 24, 177–183.

[pbi12522-bib-0025] Gupta, S. , Chakraborti, D. , Sengupta, A. , Basu, D. and Das, S. (2010) Primary metabolism of chickpea is the initial target of wound inducing early sensed *Fusarium oxysporum* f. sp *ciceri* race 1. PLoS ONE, 5, e9030. 20140256 10.1371/journal.pone.0009030 PMC2815786

[pbi12522-bib-0026] Gupta, S. , Bhar, A. and Das, S. (2013a) Understanding the molecular defense responses of host during chickpea‐Fusarium interplay: where do we stand? Funct. Plant Biol. 40, 1285–1297. 32481195 10.1071/FP13063

[pbi12522-bib-0027] Gupta, S. , Bhar, A. , Chatterjee, M. and Das, S. (2013b) *Fusarium oxysporum* f. sp *ciceri* race 1 induced redox state alterations are coupled to downstream defense signaling in root tissues of chickpea (*Cicer arietinum* L.). PLoS ONE, 8, e73163. 24058463 10.1371/journal.pone.0073163 PMC3772884

[pbi12522-bib-0028] Gurjar, G. , Barve, M. , Giri, A. and Gupta, V. (2009) Identification of Indian pathogenic races of *Fusarium oxysporum* f. sp *ciceris* with gene specific, ITS and random markers. Mycologia, 101, 484–495. 19623928 10.3852/08-085

[pbi12522-bib-0029] Gurjar, G. , Giri, A.P. and Gupta, V.S. (2012) Gene expression profiling during wilting in chickpea caused by *Fusarium oxysporum* f. sp. *ciceri* . Am. J. Plant Sci. 3, 190–201.

[pbi12522-bib-0030] Haware, M.P. , Nene, Y.L. and Natarajan, M. (1996) Survival of *Fusarium oxysporum* f. sp. *ciceri* in the soil in the absence of chickpea. Phytopathol. Mediterr. 35, 9–12.

[pbi12522-bib-0031] Heard, N.A. , Holmes, C.C. and Stephens, D.A. (2006) A quantitative study of gene regulation involved in the immune response of anopheline mosquitoes: an application of Bayesian hierarchical clustering of curves. J. Am. Stat. Assoc. 101, 18–29.

[pbi12522-bib-0032] Hodzic, A. , Rappolt, M. , Amenitsch, H. , Laggner, P. and Pabst, G. (2008) Differential modulation of membrane structure and fluctuations by plant sterols and cholesterol. Biophys. J . 94, 3935–3944. 18234811 10.1529/biophysj.107.123224 PMC2367187

[pbi12522-bib-0033] Hosel, W. and Barz, W. (1975) Beta‐glucosidases from *Cicer arietinum* L ‐ purification and properties of isoflavone‐7‐o‐glucoside specific beta‐glucosidases. Eur. J. Biochem. 57, 607–616. 240725 10.1111/j.1432-1033.1975.tb02336.x

[pbi12522-bib-0034] Isaacson, T. , Damasceno, C.M.B. , Saravanan, R.S. , He, Y. , Catala, C. , Saladie, M. and Rose, J.K.C. (2006) Sample extraction techniques for enhanced proteomic analysis of plant tissues. Nat. Protoc. 1, 769–774. 17406306 10.1038/nprot.2006.102

[pbi12522-bib-0035] Itkin, M. , Heinig, U. , Tzfadia, O. , Bhide, A.J. , Shinde, B. , Cardenas, P.D. , Bocobza, S.E. *et al*. (2013) Biosynthesis of antinutritional alkaloids in solanaceous crops is mediated by clustered genes. Science, 341, 175–179. 23788733 10.1126/science.1240230

[pbi12522-bib-0036] Jimenez‐Diaz, R.M. , Trapero‐Casas, A. and de Cabrera la Colina, J. (1989) Races of *Fusarium oxysporum* f. sp. *ciceri* infecting chickpea in southern Spain. In Vascular Wilt Diseases of Plants ( Tjamos, E.C. and Beckman, C.H. , eds), NATO ASI Series, H28, pp. 515–520. Berlin: Springer Verlag.

[pbi12522-bib-0037] Jimenez‐Fernandez, D. , Landa, B.B. , Kang, S. , Jimenez‐Diaz, R.M. and Navas‐Cortes, J.A. (2013) Quantitative and microscopic assessment of compatible and incompatible interactions between chickpea cultivars and *Fusarium oxysporum* f. sp. *ciceri* races. PLoS ONE, 8, e61360. 23613839 10.1371/journal.pone.0061360 PMC3629054

[pbi12522-bib-0038] Jobic, C. , Boisson, A.M. , Gout, E. , Rascle, C. , Fevre, M. , Cotton, P. and Bligny, R. (2007) Metabolic processes and carbon nutrient exchanges between host and pathogen sustain the disease development during sunflower infection by *Sclerotinia sclerotiorum* . Planta, 226, 251–265. 17219185 10.1007/s00425-006-0470-2

[pbi12522-bib-0039] Kawalleck, P. , Plesch, G. , Hahlbrock, K. and Somssich, I.E. (1992) Induction by fungal elicitor of s‐adenosyl‐l‐methionine synthetase and s‐adenosyl‐l‐homocysteine hydrolase messenger‐RNAs in cultured‐cells and leaves of *Petroselinum crispum* . Proc. Natl Acad. Sci. USA 89, 4713–4717. 1374911 10.1073/pnas.89.10.4713 PMC49153

[pbi12522-bib-0040] Kim, H.K. , Choi, Y.H. and Verpoorte, R. (2010) NMR‐based metabolomic analysis of plants. Nat. Protoc. 5, 536–549. 20203669 10.1038/nprot.2009.237

[pbi12522-bib-0041] Kumar, Y. , Dholakia, B.B. , Panigrahi, P. , Kadoo, N.Y. , Giri, A.P. and Gupta, V.S. (2015) Metabolic profiling of chickpea‐Fusarium interaction identifies differential modulation of disease resistance pathways. Phytochemistry, 116, 120–129. 25935544 10.1016/j.phytochem.2015.04.001

[pbi12522-bib-0042] Kumaraswamy, K.G. , Kushalappa, A.C. , Choo, T.M. , Dion, Y. and Rioux, S. (2011) Mass spectrometry based metabolomics to identify potential biomarkers for resistance in barley against fusarium head blight (*Fusarium graminearum*). J. Chem. Ecol. 37, 846–856. 21701847 10.1007/s10886-011-9989-1

[pbi12522-bib-0043] Kushalappa, A.C. and Gunnaiah, R. (2013) Metabolo‐proteomics to discover plant biotic stress resistance genes. Trends Plant Sci. 18, 522–531. 23790252 10.1016/j.tplants.2013.05.002

[pbi12522-bib-0044] Lee, J. , Feng, J. , Campbell, K.B. , Scheffler, B.E. , Garrett, W.M. , Thibivilliers, S. , Stacey, G. *et al*. (2009) Quantitative proteomic analysis of bean plants infected by a virulent and avirulent obligate rust fungus. Mol. Cell Proteomics 8, 19–31. 18755735 10.1074/mcp.M800156-MCP200

[pbi12522-bib-0045] Li, G.Z. , Vissers, J.P.C. , Silva, J.C. , Golick, D. , Gorenstein, M.V. and Geromanos, S.J. (2009) Database searching and accounting of multiplexed precursor and product ion spectra from the data independent analysis of simple and complex peptide mixtures. Proteomics, 9, 1696–1719. 19294629 10.1002/pmic.200800564

[pbi12522-bib-0046] Lionetti, V. , Raiola, A. , Camardella, L. , Giovane, A. , Obel, N. , Pauly, M. , Favaron, F. *et al*. (2007) Overexpression of pectin methylesterase inhibitors in *Arabidopsis* restricts fungal infection by *Botrytis cinerea* . Plant Physiol. 143, 1871–1880. 17277091 10.1104/pp.106.090803 PMC1851811

[pbi12522-bib-0047] Liu, C.X. , Hao, F.H. , Hu, J. , Zhang, W.L. , Wan, L.L. , Zhu, L.L. , Tang, H.R. *et al*. (2010) Revealing different systems responses to brown planthopper infestation for pest susceptible and resistant rice plants with the combined metabonomic and gene‐expression analysis. J. Proteome Res. 9, 6774–6785. 20936879 10.1021/pr100970q

[pbi12522-bib-0048] Lozovaya, V. , Ulanov, A. , Lygin, A. , Duncan, D. and Widholm, J. (2006) Biochemical features of maize tissues with different capacities to regenerate plants. Planta, 224, 1385–1399. 16941117 10.1007/s00425-006-0328-7

[pbi12522-bib-0049] Lytle, B.L. , Song, J. , de la Cruz, N.B. , Peterson, F.C. , Johnson, K.A. , Bingman, C.A. , Phillips, G.N. Jr *et al*. (2009) Structures of two *Arabidopsis thaliana* major latex proteins represent novel helix‐grip folds. Proteins, 76, 237–243. 19326460 10.1002/prot.22396 PMC2845785

[pbi12522-bib-0050] Ma, L. , Jiang, S. , Lin, G. , Cai, J. , Ye, X. , Chen, H. , Li, M. *et al*. (2013) Wound‐induced pectin methylesterases enhance banana (Musa spp. AAA) susceptibility to *Fusarium oxysporum* f. sp. *cubense* . J. Exp. Bot. 64, 2219–2229. 23580752 10.1093/jxb/ert088 PMC3654420

[pbi12522-bib-0051] Maere, S. , Heymans, K. and Kuiper, M. (2005) BiNGO: a Cytoscape plugin to assess overrepresentation of gene ontology categories in biological networks. Bioinformatics, 21, 3448–3449. 15972284 10.1093/bioinformatics/bti551

[pbi12522-bib-0052] Masuta, C. , Tanaka, H. , Uehara, K. , Kuwata, S. , Koiwai, A. and Noma, M. (1995) Broad resistance to plant viruses in transgenic plants conferred by antisense inhibition of a host gene essential in S‐adenosylmethionine‐dependent transmethylation reactions. Proc. Natl Acad. Sci. USA 92, 6117–6121. 11607550 10.1073/pnas.92.13.6117 PMC41653

[pbi12522-bib-0053] MauchMani, B. and Slusarenko, A.J. (1996) Production of salicylic acid precursors is a major function of phenylalanine ammonia‐lyase in the resistance of arabidopsis to *Peronospora parasitica* . Plant Cell, 8, 203–212. 12239383 10.1105/tpc.8.2.203 PMC161092

[pbi12522-bib-0054] Maytalman, D. , Mert, Z. , Baykal, A.T. , Inan, C. , Gunel, A. and Hasancebi, S. (2013) Proteomic analysis of early responsive resistance proteins of wheat (*Triticum aestivum*) to yellow rust (*Puccinia striiformis* f. sp *tritici*) using ProteomeLab PF2D. Plant Omics, 6, 24–35.

[pbi12522-bib-0055] Mehta, A. , Brasileiro, A.C.M. , Souza, D.S.L. , Romano, E. , Campos, M.A. , Grossi‐De‐Sa, M.F. , Silva, M.S. *et al*. (2008) Plant‐pathogen interactions: what is proteomics telling us? FEBS J. 275, 3731–3746. 18616468 10.1111/j.1742-4658.2008.06528.x

[pbi12522-bib-0056] Naoumkina, M. , Farag, M.A. , Sumner, L.W. , Tang, Y.H. , Liu, C.J. and Dixon, R.A. (2007) Different mechanisms for phytoalexin induction by pathogen and wound signals in *Medicago truncatula* . Proc. Natl Acad. Sci. USA 104, 17909–17915. 17971436 10.1073/pnas.0708697104 PMC2084270

[pbi12522-bib-0057] Nelson, D.E. , Repetti, P.P. , Adams, T.R. , Creelman, R.A. , Wu, J. , Warner, D.C. , Anstrom, D.C. *et al*. (2007) Plant nuclear factor Y (NF‐Y) B subunits confer drought tolerance and lead to improved corn yields on water‐limited acres. Proc. Natl Acad. Sci. USA 104, 16450–16455. 17923671 10.1073/pnas.0707193104 PMC2034233

[pbi12522-bib-0058] Nimbalkar, S.B. , Harsulkar, A.M. , Giri, A.P. , Sainani, M.N. , Franceschi, V. and Gupta, V.S. (2006) Differentially expressed gene transcripts in roots of resistant and susceptible chickpea plant (*Cicer arietinum* L.) upon *Fusarium oxysporum* infection. Physiol. Mol. Plant Pathol. 68, 176–188.

[pbi12522-bib-0059] Patel, V.J. , Thalassinos, K. , Slade, S.E. , Connolly, J.B. , Crombie, A. , Murrell, J.C. and Scrivens, J.H. (2009) A comparison of labeling and label‐free mass spectrometry‐based proteomics approaches. J. Proteome Res. 8, 3752–3759. 19435289 10.1021/pr900080y

[pbi12522-bib-0060] Qiang, X.Y. , Zechmann, B. , Reitz, M.U. , Kogel, K.H. and Schafer, P. (2012) The mutualistic fungus *Piriformospora indica* colonizes arabidopsis roots by inducing an endoplasmic reticulum stress‐triggered caspase‐dependent cell death. Plant Cell, 24, 794–809. 22337916 10.1105/tpc.111.093260 PMC3315247

[pbi12522-bib-0061] Raju, S. , Jayalakshmi, S.K. and Sreeramulu, K. (2008) Comparative studies on induction of defense related enzymes in two different cultivars of chickpea (*Cicer arietinum* L) genotypes by salicylic acid, spermine and *Fusarium oxysporum* f. sp. *ciceri* . Aust. J. Crop Sci. 2, 121–140.

[pbi12522-bib-0062] Rea, G. , Laurenzi, M. , Tranquilli, E. , D'Ovidio, R. , Federico, R. and Angelini, R. (1998) Developmentally and wound‐regulated expression of the gene encoding a cell wall copper amine oxidase in chickpea seedlings. FEBS Lett. 437, 177–182. 9824285 10.1016/s0014-5793(98)01219-8

[pbi12522-bib-0063] Rea, G. , Metoui, O. , Infantino, A. , Federico, R. and Angelini, R. (2002) Copper amine oxidase expression in defense responses to wounding and *Ascochyta rabiei* invasion. Plant Physiol. 128, 865–875. 11891243 10.1104/pp.010646 PMC152200

[pbi12522-bib-0064] Roberts, M.R. (2003) 14‐3‐3 Proteins find new partners in plant cell signalling. Trends Plant Sci. 8, 218–223. 12758039 10.1016/S1360-1385(03)00056-6

[pbi12522-bib-0065] Sawada, K. , Hasegawa, M. , Tokuda, L. , Kameyama, J. , Kodama, O. , Kohchi, T. , Yoshida, K. *et al*. (2004) Enhanced resistance to blast fungus and bacterial blight in transgenic rice constitutively expressing OsSBP, a rice homologue of mammalian selenium‐binding proteins. Biosci. Biotechnol. Biochem. 68, 873–880. 15118317 10.1271/bbb.68.873

[pbi12522-bib-0066] Scalet, M. , Federico, R. and Angelini, R. (1991) Time courses of diamine oxidase and peroxidase‐activities, and polyamine changes after mechanical injury of chickpea seedlings. J. Plant Physiol. 137, 571–575.

[pbi12522-bib-0068] Shetty, N.P. , Jensen, J.D. , Knudsen, A. , Finnie, C. , Geshi, N. , Blennow, A. , Collinge, D.B. *et al*. (2009) Effects of beta‐1,3‐glucan from *Septoria tritici* on structural defence responses in wheat. J. Exp. Bot. 60, 4287–4300. 19880540 10.1093/jxb/erp269

[pbi12522-bib-0069] Subramanian, S. , Graham, M.Y. , Yu, O. and Graham, T.L. (2005) RNA interference of soybean isoflavone synthase genes leads to silencing in tissues distal to the transformation site and to enhanced susceptibility to *Phytophthora sojae* . Plant Physiol. 137, 1345–1353. 15778457 10.1104/pp.104.057257 PMC1088325

[pbi12522-bib-0070] Tavernier, V. , Cadiou, S. , Pageau, K. , Lauge, R. , Reisdorf‐Cren, M. , Langin, T. and Masclaux‐Daubresse, C. (2007) The plant nitrogen mobilization promoted by *Colletotrichum lindemuthianum* in Phaseolus leaves depends on fungus pathogenicity. J. Exp. Bot. 58, 3351–3360. 17977849 10.1093/jxb/erm182

[pbi12522-bib-0071] Trygg, J. (2002) O2‐PLS for qualitative and quantitative analysis in multivariate calibration. J. Chemom. 16, 283–293.

[pbi12522-bib-0072] Wang, Y. , Yang, L.M. , Xu, H.B. , Li, Q.F. , Ma, Z.Q. and Chu, C.G. (2005) Differential proteomic analysis of proteins in wheat spikes induced by *Fusarium graminearum* . Proteomics, 5, 4496–4503. 16222720 10.1002/pmic.200401317

[pbi12522-bib-0073] Wang, Y.L. , Lawler, D. , Larson, B. , Ramadan, Z. , Kochhar, S. , Holmes, E. and Nicholson, J.K. (2007) Metabonomic investigations of aging and caloric restriction in a life‐long dog study. J. Proteome Res. 6, 1846–1854. 17411081 10.1021/pr060685n

[pbi12522-bib-0074] Ward, E.R. , Payne, G.B. , Moyer, M.B. , Williams, S.C. , Dincher, S.S. , Sharkey, K.C. , Beck, J.J. *et al*. (1991) Differential regulation of beta‐1,3‐glucanase messenger‐RNAs in response to pathogen infection. Plant Physiol. 96, 390–397. 16668198 10.1104/pp.96.2.390 PMC1080782

[pbi12522-bib-0075] Xiao, C.N. , Dai, H. , Liu, H.B. , Wang, Y.L. and Tang, H.R. (2008) Revealing the metabonomic variation of rosemary extracts using H^1^ NMR spectroscopy and multivariate data analysis. J. Agric. Food Chem. 56, 10142–10153. 18800806 10.1021/jf8016833

[pbi12522-bib-0076] Yang, F. , Jensen, J.D. , Svensson, B. , Jorgensen, H.J.L. , Collinge, D.B. and Finnie, C. (2010) Analysis of early events in the interaction between *Fusarium graminearum* and the susceptible barley (*Hordeum vulgare*) cultivar Scarlett. Proteomics, 10, 3748–3755. 20925056 10.1002/pmic.201000243

[pbi12522-bib-0077] Ye, C.M. , Dickman, M.B. , Whitham, S.A. , Payton, M. and Verchot, J. (2011) The unfolded protein response is triggered by a plant viral movement protein. Plant Physiol. 156, 741–755. 21474436 10.1104/pp.111.174110 PMC3177272

[pbi12522-bib-0078] Zhou, W.C. , Eudes, F. and Laroche, A. (2006) Identification of differentially regulated proteins in response to a compatible interaction between the pathogen *Fusarium graminearum* and its host, *Triticum aestivum* . Proteomics, 6, 4599–4609. 16858732 10.1002/pmic.200600052

