# Supplementary material for: Fusarium oxysporum mediates systems metabolic reprogramming of chickpea roots as revealed by a combination of proteomics and metabolomics
Source: Plant Biotechnol J. 2016 Jan 23;14(7):1589–603. doi: 10.1111/pbi.12522 (PMC5066658; doi:10.1111/pbi.12522)
Supplement: Supplementary file 1 — Figure S1 The Foc resistant‐DV and susceptible‐JG62 (JG) chickpea plants at early and late stages after inoculation. [file PBI-14-1589-s008.pptx]

## Slide 1
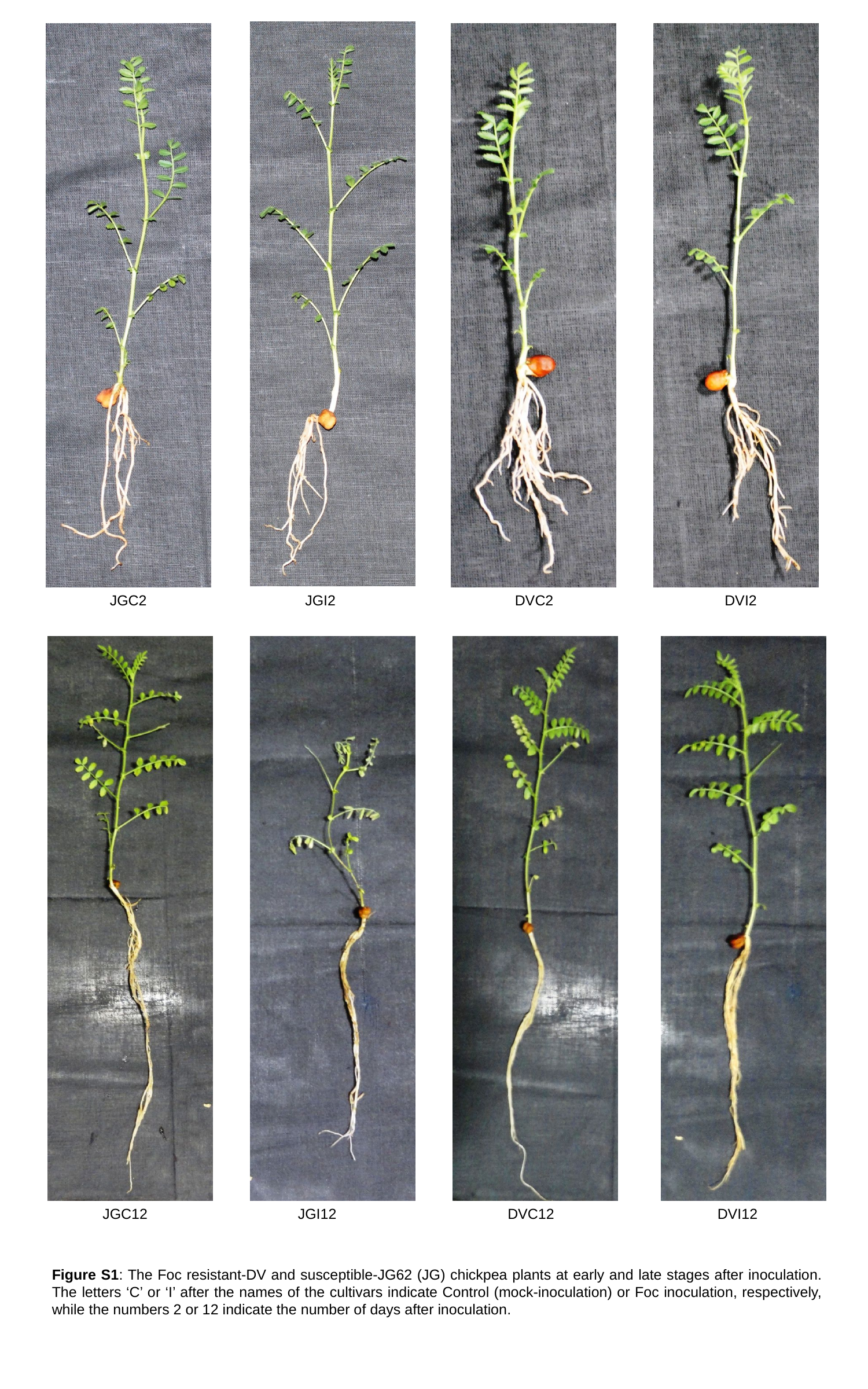

JGC2
JGI2
DVC2
DVI2
JGC12
JGI12
DVC12
DVI12
Figure S1: The Foc resistant-DV and susceptible-JG62 (JG) chickpea plants at early and late stages after inoculation. The letters ‘C’ or ‘I’ after the names of the cultivars indicate Control (mock-inoculation) or Foc inoculation, respectively, while the numbers 2 or 12 indicate the number of days after inoculation.
