# Supplementary material for: Fusarium oxysporum mediates systems metabolic reprogramming of chickpea roots as revealed by a combination of proteomics and metabolomics
Source: Plant Biotechnol J. 2016 Jan 23;14(7):1589–603. doi: 10.1111/pbi.12522 (PMC5066658; doi:10.1111/pbi.12522)
Supplement: Supplementary file 2 — Figure S2 Permutation test results for OPLS‐DA models with two components and 200 permutations. [file PBI-14-1589-s007.pptx]

## Slide 1
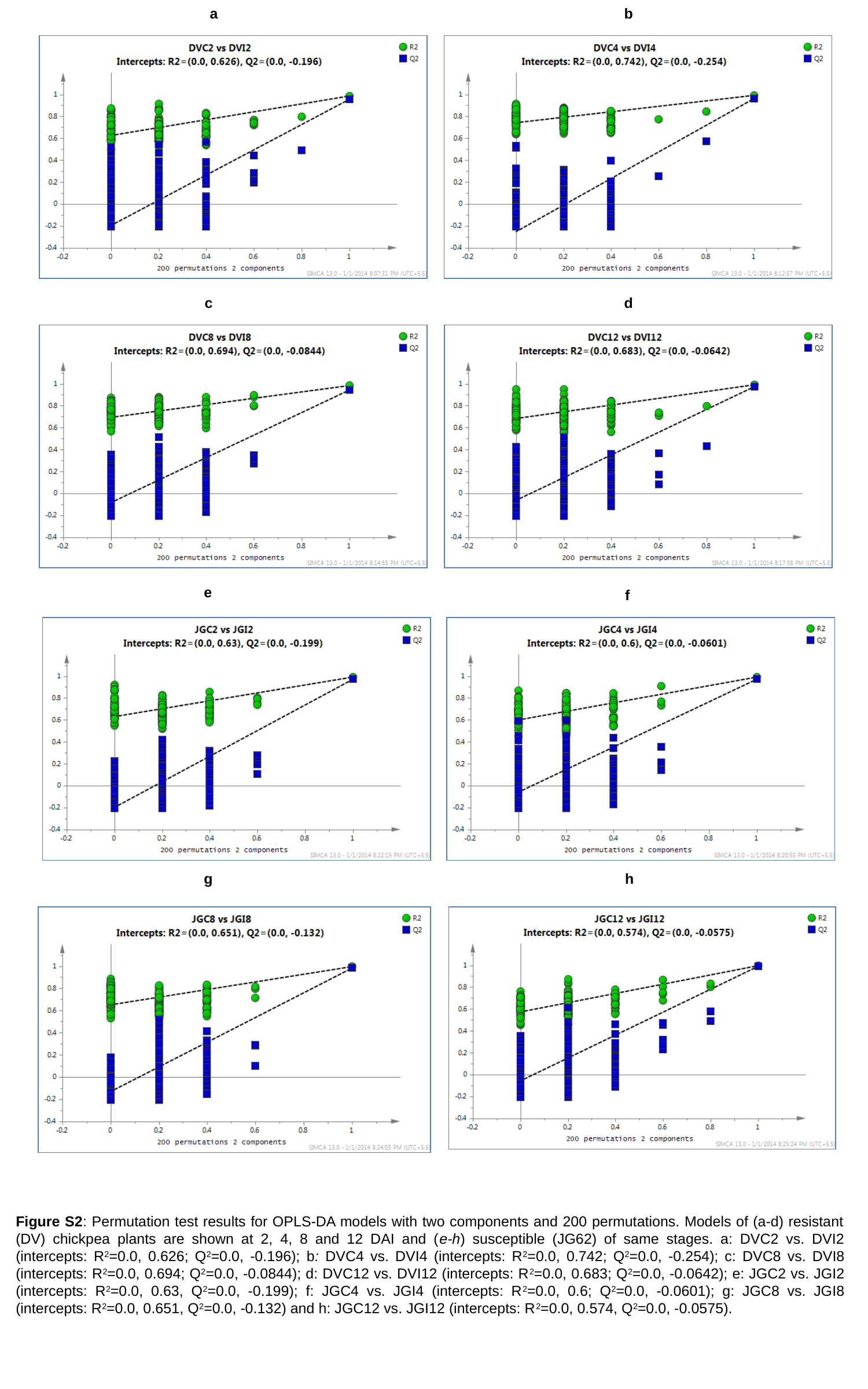

a
b
c
d
e
f
g
h
Figure S2: Permutation test results for OPLS-DA models with two components and 200 permutations. Models of (a-d) resistant (DV) chickpea plants are shown at 2, 4, 8 and 12 DAI and (e-h) susceptible (JG62) of same stages. a: DVC2 vs. DVI2 (intercepts: R2=0.0, 0.626; Q2=0.0, -0.196); b: DVC4 vs. DVI4 (intercepts: R2=0.0, 0.742; Q2=0.0, -0.254); c: DVC8 vs. DVI8 (intercepts: R2=0.0, 0.694; Q2=0.0, -0.0844); d: DVC12 vs. DVI12 (intercepts: R2=0.0, 0.683; Q2=0.0, -0.0642); e: JGC2 vs. JGI2 (intercepts: R2=0.0, 0.63, Q2=0.0, -0.199); f: JGC4 vs. JGI4 (intercepts: R2=0.0, 0.6; Q2=0.0, -0.0601); g: JGC8 vs. JGI8 (intercepts: R2=0.0, 0.651, Q2=0.0, -0.132) and h: JGC12 vs. JGI12 (intercepts: R2=0.0, 0.574, Q2=0.0, -0.0575).
