# Supplementary material for: Fusarium oxysporum mediates systems metabolic reprogramming of chickpea roots as revealed by a combination of proteomics and metabolomics
Source: Plant Biotechnol J. 2016 Jan 23;14(7):1589–603. doi: 10.1111/pbi.12522 (PMC5066658; doi:10.1111/pbi.12522)
Supplement: Supplementary file 3 — Figure S3 Pattern of lignification in cross sections of chickpea root tissue from resistant‐DV and susceptible‐JG62 at different Foc inoculation stages using phloroglucinol/hydrochloric acid stain. [file PBI-14-1589-s005.pptx]

## Slide 1
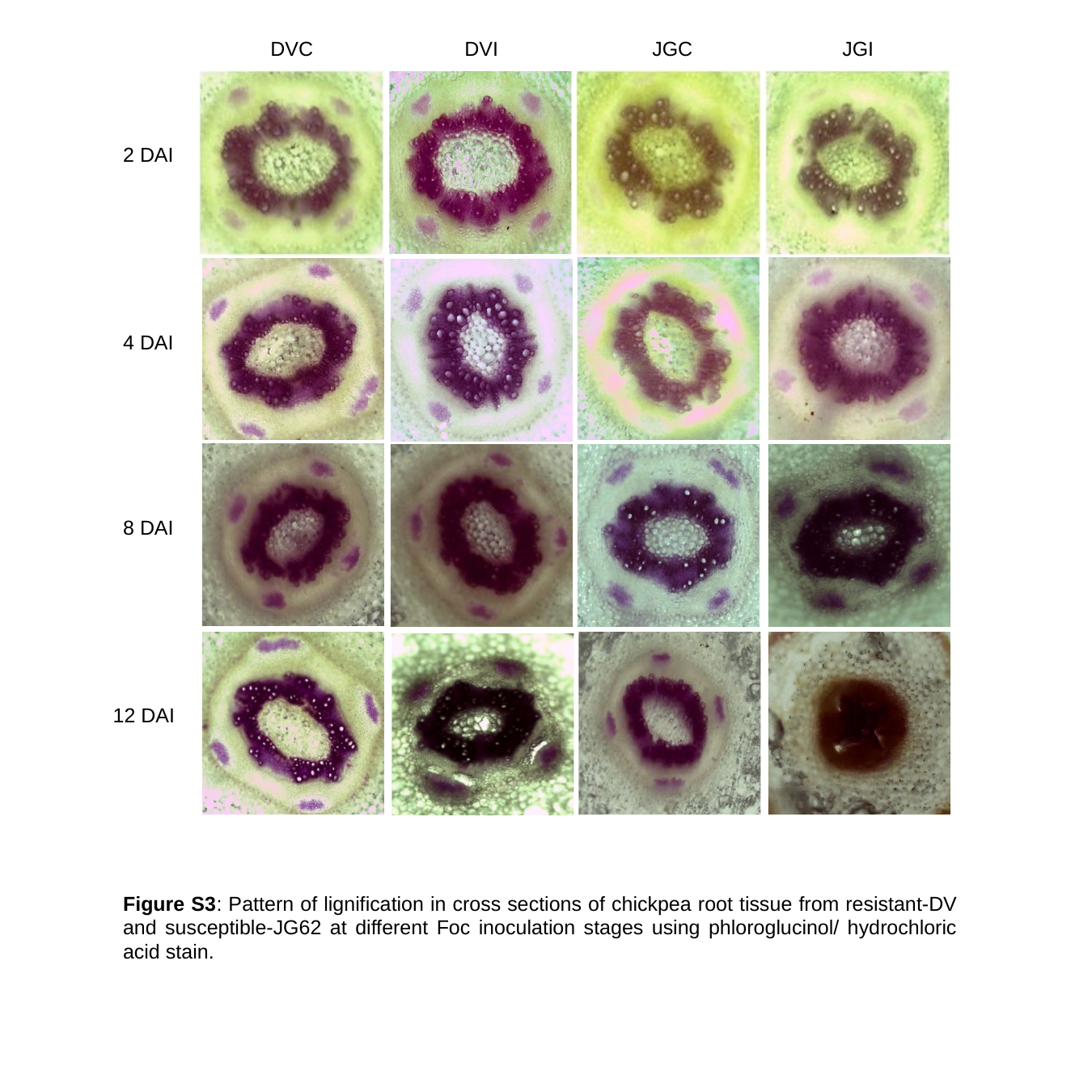

DVC
DVI
JGC
JGI
2 DAI
4 DAI
8 DAI
12 DAI
Figure S3: Pattern of lignification in cross sections of chickpea root tissue from resistant-DV and susceptible-JG62 at different Foc inoculation stages using phloroglucinol/ hydrochloric acid stain.
