# Supplementary material for: Fusarium oxysporum mediates systems metabolic reprogramming of chickpea roots as revealed by a combination of proteomics and metabolomics
Source: Plant Biotechnol J. 2016 Jan 23;14(7):1589–603. doi: 10.1111/pbi.12522 (PMC5066658; doi:10.1111/pbi.12522)
Supplement: Supplementary file 4 — Figure S4 Design of experiment with details of sample preparation, tissue collection stages, approaches and data analysis. [file PBI-14-1589-s009.pptx]

## Slide 1
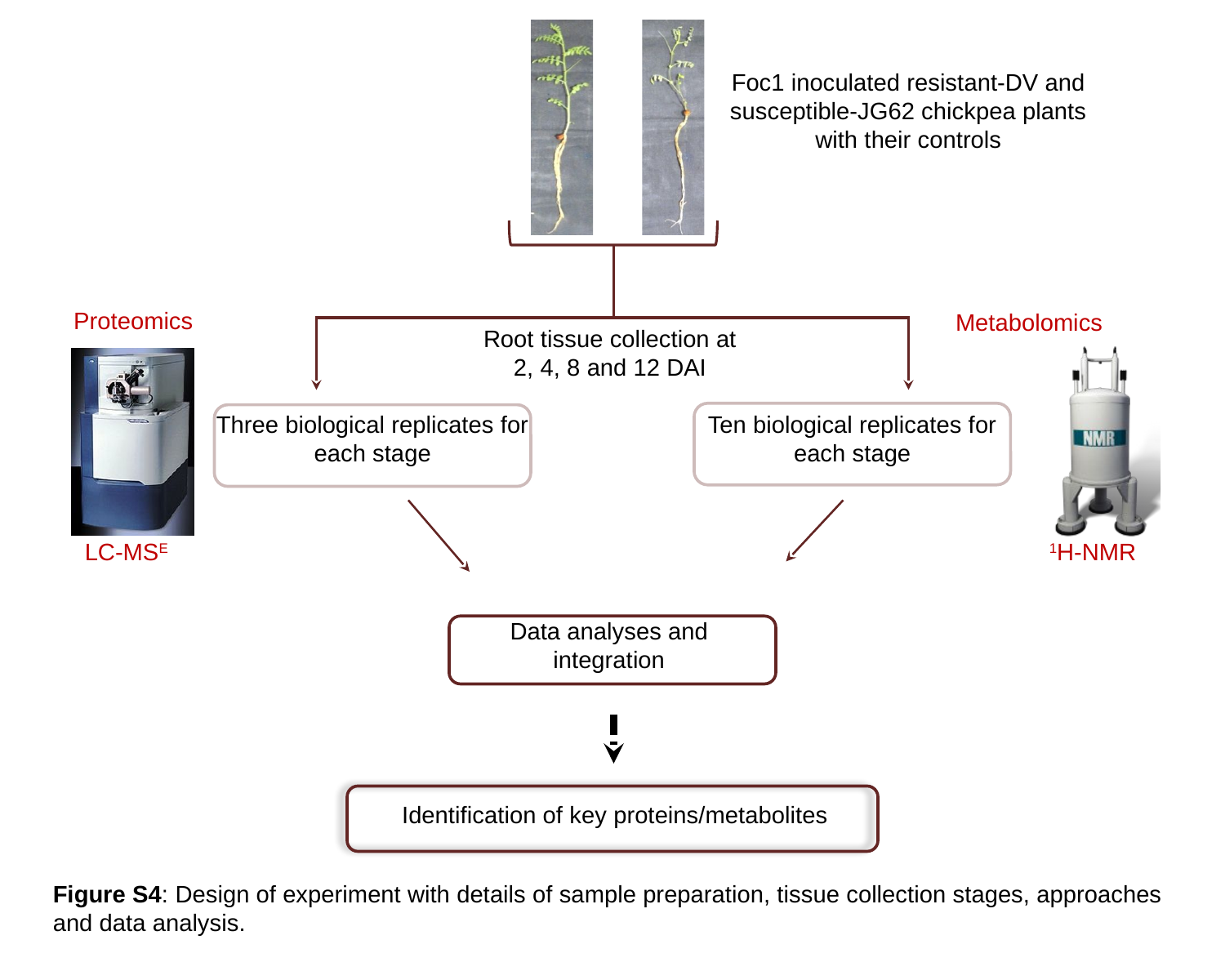

Foc1 inoculated resistant-DV and susceptible-JG62 chickpea plants with their controls
Proteomics
Metabolomics
Root tissue collection at
2, 4, 8 and 12 DAI
Ten biological replicates for each stage
Three biological replicates for each stage
LC-MSE
1H-NMR
Data analyses and integration
Identification of key proteins/metabolites
Figure S4: Design of experiment with details of sample preparation, tissue collection stages, approaches and data analysis.
