# Supplementary material for: Fusarium oxysporum mediates systems metabolic reprogramming of chickpea roots as revealed by a combination of proteomics and metabolomics
Source: Plant Biotechnol J. 2016 Jan 23;14(7):1589–603. doi: 10.1111/pbi.12522 (PMC5066658; doi:10.1111/pbi.12522)
Supplement: Supplementary file 5 — Figure S5 Protein quality control measurements. [file PBI-14-1589-s001.pptx]

## Slide 1
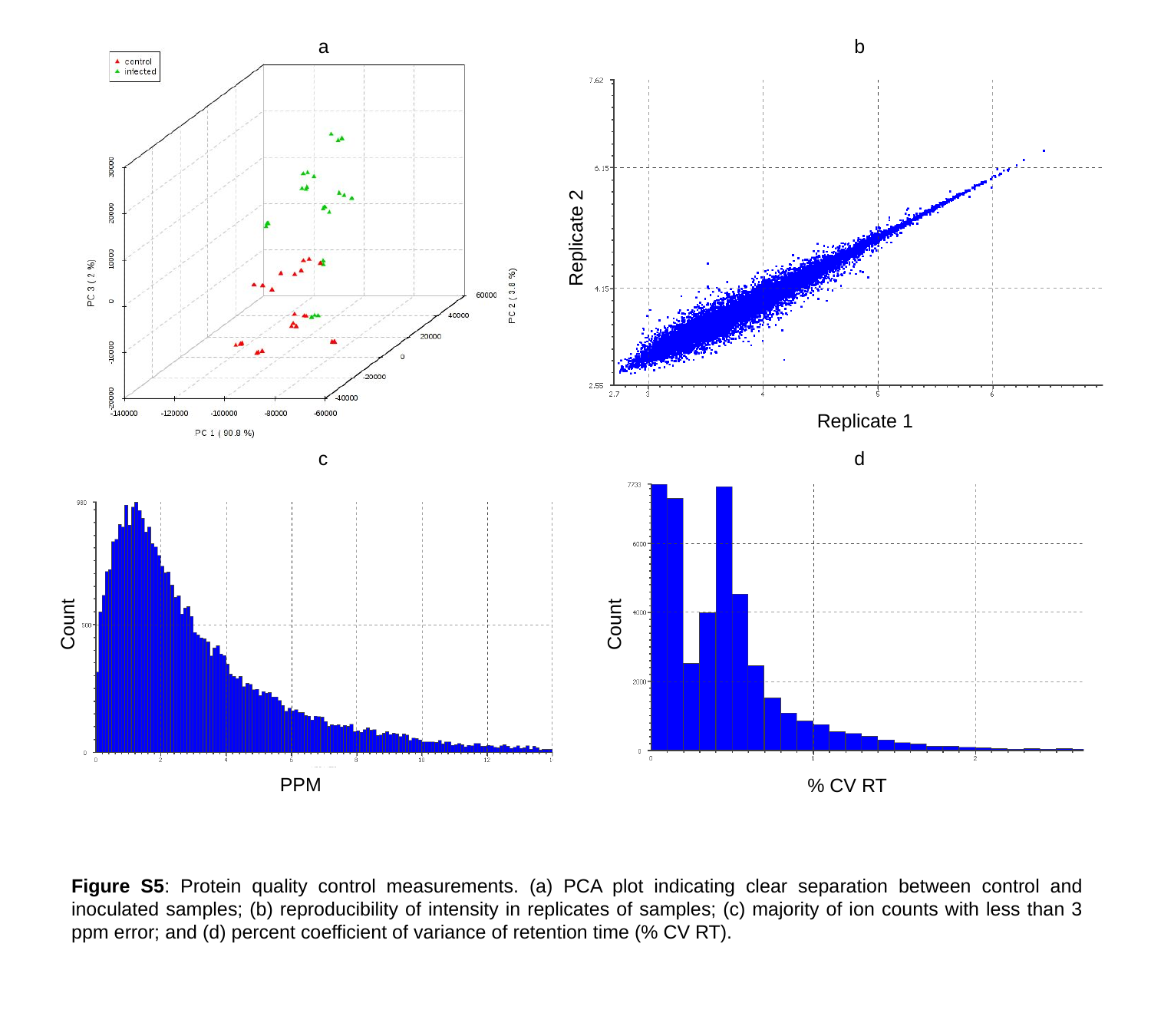

a
b
Replicate 1
Replicate 2
c
PPM
d
Count
% CV RT
Count
Figure S5: Protein quality control measurements. (a) PCA plot indicating clear separation between control and inoculated samples; (b) reproducibility of intensity in replicates of samples; (c) majority of ion counts with less than 3 ppm error; and (d) percent coefficient of variance of retention time (% CV RT).
