# Supplementary material for: Fusarium oxysporum mediates systems metabolic reprogramming of chickpea roots as revealed by a combination of proteomics and metabolomics
Source: Plant Biotechnol J. 2016 Jan 23;14(7):1589–603. doi: 10.1111/pbi.12522 (PMC5066658; doi:10.1111/pbi.12522)
Supplement: Supplementary file 7 — Table S2 Assignments of metabolites from 1H‐NMR analysis. [file PBI-14-1589-s003.doc]

**Supplemental Table S2: Assignments of metabolites from ^1^H-NMR analysis**

| **Key** | **Metabolites** | **Assignment** | **δ^1^ H (multiplicity)^a^** | **δ^13^ C** | **Assigned with** |
| --- | --- | --- | --- | --- | --- |
| 1 | Phytosterol | CH_3_ | 0.74(s), 0.78(s), 0.83(s), 0.86 (s) | 23.1, 21.9, 17.7, 15.8 | HSQC, HMBC |
| 2 | Isoleucine | αCH | 3.68(d) | 62.6 | JRE, COSY, TOCSY, HSQC, HMBC |
|  |  | βCH | 1.98(m) | 39.4 |  |
|  |  | γCH_2_ | 1.24(m), 1.45(m) | 27.4 | JRE, COSY, TOCSY, HMBC |
|  |  | γ’CH_3_ | 1.02(d, 7.0Hz) | 17.6 | JRE, COSY, TOCSY, HSQC, HMBC |
|  |  | δCH_3_ | 0.94(t, 7.3Hz) | 13.9 |  |
| 3 | Leucine | βCH_2_ | 1.73(m) | 42.4 | JRE, COSY, TOCSY, HMBC, HSQC |
|  |  | γCH | 1.69(m) | 27.4 |  |
|  |  | δCH_3_ | 0.98(d, 6.5Hz) | 24.5 | JRE, COSY, TOCSY, HSQC |
|  |  | δ’CH_3_ | 0.96(d, 5.9Hz) | 24.5 |  |
| 4 | Valine | αCH | 3.62(d, 4.3Hz) | 63.4 | JRE, COSY, TOCSY, HSQC, HMBC |
|  |  | βCH | 2.28(m) | 32.1 | JRE, COSY, TOCSY, HSQC, HMBC |
|  |  | γCH_3_ | 1.01(d, 6.9Hz) | 19.6 | JRE, COSY, TOCSY, HSQC, HMBC |
|  |  | γ’CH_3_ | 1.06(d, 6.9Hz) | 20.9 |  |
|  |  | COOH |  | 177.3 | HMBC |
| 5 | D-3-hydroxybutyrate | CH | 4.16(dt) | 68.4 | HMBC |
|  |  | CH_2_ | 2.41(dd) | 46.5 | COSY, TOCSY |
|  |  | CH_2_ | 2.31(dd) | 46.5 |  |
|  |  | γCH_3_ | 1.20(d, 10.2Hz) | 24.6 | JRE, COSY, TOCSY, HSQC |
| 6 | Lipid | CH_3_ | 0.88(s) | 19.3 | TOCSY, HSQC, HMBC |
|  |  | (CH_2_)_n_ | 1.29(m), 1.59(m) | 20.1 |  |
| 7 | Lactate | αCH | 4.13(q) | # | JRE, COSY, TOCSY |
|  |  | βCH_3_ | 1.34(d, 6.56Hz) | 22.9 | JRE, COSY, TOCSY, HSQC |
|  |  | COOH |  | 183.1 | HMBC |
| 8 | Threonine | α-CH | 3.53(d, 2.91Hz) | 63.5 | JRE, COSY, |
|  |  | β-CH2 | 4.23(m) | 69.1 | TOCSY,HSQC, HMBC |
|  |  | γ-CH3 | 1.34(d. 6.5Hz) | 22.9 |  |
|  |  | COOH |  | # |  |
| 9 | Alanine | αCH | 3.79(q) | 53.4 | JRE, COSY, TOCSY,HSQC, HMBC |
|  |  | βCH_3_ | 1.49(d, 7.3Hz) | 19.1 |  |
|  |  | COOH |  | # | HMBC |
| 10 | Lysine | αCH | 3.73(t) | 57.4 | JRE, COSY, TOCSY, HSQC, HMBC |
|  |  | βCH_2_ | 1.91(m) | # |  |
|  |  | γCH_2_ | 1.49(m) | 25.8 |  |
|  |  | εCH_2_ | 1.73(m) | 29.0 |  |
|  |  | εCH_2_ | 3.01(t, 7.5Hz) | 42.2 |  |
|  |  | COOH |  | # | HMBC |
| 11 | Acetate | CH_3_ | 1.94(s) | 25.7 | JRE, HSQC, HMBC |
|  |  | COOH |  | # |  |
| 12 | Acetamide | CH_3_ | 2.03(s) | 17.6 | JRE, HSQC |
|  |  | CO |  | # | HMBC |
| 12 | Glutamate | αCH | 3.77(m) | 57.6 | JRE, COSY, TOCSY, HSQC, HMBC |
|  |  | βCH_2_ | 2.06(m), 2.1(m) | 29.8 |  |
|  |  | γCH_2_ | 2.34(dt, 3.8, 11.6Hz) | 36.4 |  |
|  |  | δCO |  | 184.3 | HMBC |
|  |  | COOH |  | 177.2 |  |
| 13 | Glutamine | αCH | 2.15(m) | 29.6 |  |
|  |  | βCH_2_ | 2.40(m) | 36.9 |  |
|  |  | γCH_2_ | 3.77(m) | 57.1 |  |
| 14 | γ-Aminobutyrate | αCH_2_ | 2.19(t, 7.6Hz) | 32.2 | JRE, COSY, TOCSY, HSQC |
|  |  | βCH_2_ | 1.9(m) | 26.0 | JRE, COSY, TOCSY, HSQC, HMBC |
|  |  | γCH_2_ | 3.12(t) | 44.2 |  |
|  |  | COOH |  | 186.1 | HMBC |
| 15 | Pyruvate | CH_3_ | 2.35(s) | 28.3 | HSQC |
|  |  | CO |  | # |  |
|  |  | COOH |  | # |  |
| 16 | Aspargine | αCH | 3.94(dd) | 54.7 | JRE, COSY, TOCSY, HSQC, HMBC |
|  |  | βCH_2_ | 2.84(dd, 8.3Hz, ) 2.94(dd, 4.01Hz) | 37.5 |  |
|  |  | CO |  | 176.5 | HMBC |
|  |  | COOH |  | 176.5 |  |
| 17 | Choline | N(CH_3_)_3_ | 3.21(s) | 56.5 | JRE, COSY, TOCSY, HSQC, HMBC |
|  |  | OCH_2_ | # | # |  |
|  |  | NCH_2_ | 3.51(m) | 70.4 | HMBC |
| 18 | Ethanolamine | αCH_2_ | 3.12 (t) | 44.3 | JRE, COSY, TOCSY, HSQC, HMBC |
|  |  | βCH_2_ | 3.81(t) | 63.6 |  |
| 19 | phosphocholine | N(CH_3_)_3_ | 3.23(s) | 56.5 | TOCSY, HSQC |
|  |  | OCH_2_ | # | # |  |
|  |  | NCH_2_ | 3.61(m) | # |  |
| 20 | Citrate | CH_2_ | 2.54(d,16.5Hz) | 47.1 | TOCSY, HSQC, HMBC |
|  |  | CH_2_ | 2.68(d, 16.5Hz) | 78.2 |  |
| 21 | Succinate | CH_2_ | 2.49(s) | 35.2 | JRE, HSQC |
|  |  | COOH |  | 182.2 | HMBC |
| 22 | β-Glucose | C_1_H | 4.59(d, 7.8Hz) | 99.2 | JRE, COSY, TOCSY, HSQC, HMBC |
|  |  | C_2_H | 3.25(t, 9.3Hz) | 77.6 |  |
|  |  | C_3_H | 3.44(dd) | 79.0 |  |
|  |  | C_4_H | 3.38(dd) | 56.1 |  |
|  |  | C_5_H | 3.41(t) | 72.8 |  |
|  |  | C_6_H | 3.73(dd), 3.83(dd) | 63.1 |  |
| 23 | α-Glucose | C_1_H | 5.12(d, 3.9Hz) | 95.4 | JRE, COSY, TOCSY, HSQC |
|  |  | C_2_H | 3.54(#) | 72.2 | COSY, TOCSY, HSQC, HMBC |
|  |  | C_3_H | 3.71(#) | 76.0 |  |
|  |  | C_4_H | 3.43(#) | 72.8 |  |
|  |  | C_5_H | 3.84(#) | 74.5 |  |
|  |  | C_6_H | 3.73(#) | 64.2 |  |
| 24 | Methanol | CH_3_ | 3.34(s) | 52.1 | JRE, HSQC |
| 25 | Malate | CH_2_ | 2.50(dd) | 44.4 | JRE, TOCSY, HSQC, HMBC |
|  |  | CH_2_ | 2.73(dd, 9.2Hz, 9.2Hz) | 44.4 |  |
|  |  | CH | 4.29(dd) | 72.5 |  |
| 26 | Sucrose | C_1_H | 3.66(s) | 64.9 | JRE, TOCSY, HSQC, HMBC |
|  |  | C_2_H | 3.80(m) | 63.6 |  |
|  |  | C_3_H | 3.81(m) | 84.9 |  |
|  |  | C_4_H | # | 79.6 |  |
|  |  | C_5_H | 5.41(d) | 95.4 |  |
|  |  |  | # | 106.7 | HMBC |
| 27 | Fructose | C_1_H | 3.80(m) | 63.6 | JRE, TOCSY, HSQC, HMBC |
|  |  | C_2_H | 3.84(m) | 84.6 |  |
|  |  | C_3_H | 4.03 (dd) | 77.2 |  |
|  |  | C_4_H | 4.17(d) | 79.8 |  |
| 28 | Trehalose | C_1_H | 3.48(d) | # | TOCSY, HSQC, HMBC |
|  |  | C_2_H | 3.69(d) | # |  |
|  |  | C_3_H | 3.80 (m) | 75.4 |  |
|  |  | C_4_H | 5.16(d) | 95.4 |  |
| 29 | D-Methanol | CH_3_ | 3.32(m) | 51.4 | HSQC |
| 30 | Uridine | C_1_ |  | 169.4 | HMBC |
|  |  | C_2_ |  | 155.0 |  |
|  |  | C_3_ | 7.88(d, 8.1Hz) | 145.1 | JRE, COSY, TOCSY, HSQC, HMBC |
|  |  | C_4_ | 5.91(d, 3.9Hz) | 104.7 |  |
|  |  | C_5_ | 5.93(d) | 104.7 | JRE, HMBC |
| 31 | 5’-CMP | C_1_H | 7.85(d, 7.5Hz) | 144.9 | JRE, COSY, TOCSY, HSQC, HMBC |
|  |  | C_2_H | 6.06(d, 6.9Hz) | 99.5 |  |
|  |  | C_3_ |  | 169.1 | HMBC |
|  |  | C_4_ |  | 160.5 |  |
| 32 | U |  | 5.64, 3.70, 4.34 | 81.6, 66.7 |  |
| 33 | U |  | 5.61, 3.66, 4.32 | 81.8 |  |
| 34 | U |  | 3.16 | 45.4, 178.6 |  |
| 35 | U |  | 6.32, 6.48 | 102.3, 97.5 | HSQC, HMBC |
| 36 | Genistein | CH  CH  CH  CH  CH | 6.51(d, 2.1Hz)  6.34 (d, 2.1Hz)  8.126 (s)  7.39 (s)  5.41(d, 3.8Hz) | 96.2 | HSQC  Spiking |
| 37 | Fumarate | CH | 6.53(s) | 138.7 | JRE, HSQC |
|  |  | COOH |  | 177.5 | HMBC |
| 38 | Luteolin | C_4_H | 6.56(d, 2.19Hz) | 108.6 | JRE, TOCSY,HSQC  Spiking |
|  |  | C_3_H | 6.78(d, 2.46Hz) | # |  |
|  |  | C_2_H | 7.09(d, 8.7Hz) | # |  |
|  |  | C_1_H | 7.45(d, 8.7Hz) | # |  |
| 39 | Orotate | CH_3_, CH  CH, CH, | 1.18(t), 3.62(m), 3.71(s),  4.82(s), 6.1(s), 6.24(s), | 101.7 | HSQC  Spiking |
| 40 | Salicin*^b^* | CH | 7.28(d) | 128.3 | JRE, HSQC |
|  |  | CH | 4.68(q) | 103.7 | HSQC |
| 41 | Clotrimazole | CH | 7.02(d, 8.7Hz) | 117.1 | JRE, TOCSY,HSQC  Spiking |
|  |  | CH  CH  CH | 7.44(d, 8.7Hz)  7.16(dd)  3.60(s), 3.644(s)  3.72(t)  8.08(d, 9.0Hz) | 133.5 |  |
| 42 | U |  | 6.82(m) | 113.9 |  |
|  |  |  | 7.46 | # |  |
| 43 | Tryptophan | C4H,ring | 7.74(d,8.28Hz) | # | JRE, COSY, TOCSY, |
|  |  | C5H,ring | # | # |  |
|  |  | C6H,ring | 7.09(t,7.37) | # |  |
|  |  | C7H,ring | 7.46(d) | # |  |
| 44 | U |  |  |  |  |
| 45 | Nicotinate*^b^* | 4CH | 8.06(d) | # |  |
| 46 | Guanidoacetate*^b^* | \ | 3.86(s) | 160.2 | JRE, HSQC |
| 47 | Quinone*^b^* | CH | 6.88(s) | # | JRE, TOCSY |
| 48 | 4-Nitrophenol | CH | 8.116(s) | 126.0 | JRE,TOCSY, HMBC |
|  |  | CH | 6.86(s) | # |  |
| 49 | Hypoxanthine | C_2_H | 8.19(s) | # | JRE, TOCSY, HSQC, HMBC |
|  |  | C_7_H | 8.21(s) | 139.7 |  |
| 50 | Adenosine | C_1_H | 8.34(s) | 143.4 | HSQC, HMBC |
|  |  | C_4_H | 8.25(s) | # | HSQC |
|  |  | C_6_H | 6.07(d, 5.9Hz) | 91.4 | JRE, COSY, TOCSY, HSQC, HMBC |
| 51 | Inosine | CH, CH,  CH, | 8.28(s), 3.91(d, 3.06 Hz),  6.05(d, 6.0Hz) | # | JRE  Spiking |
| 52 | Formate | CH | 8.46(s) | # | JRE |

^a^ Multiplicity: s-singlet; d- doublet; dd, doublet of doublets; t- triplet; q- quartet; U- unidentified signal; #- signals or multiplicities were not determined.

^b^ tentatively assigned

Confirmed assignments for the metabolites with spiking are labeled in red while other metabolites (without spiking) are in black.
