# Supplementary material for: Fusarium oxysporum mediates systems metabolic reprogramming of chickpea roots as revealed by a combination of proteomics and metabolomics
Source: Plant Biotechnol J. 2016 Jan 23;14(7):1589–603. doi: 10.1111/pbi.12522 (PMC5066658; doi:10.1111/pbi.12522)
Supplement: Supplementary file 8 — Table S3 Metabolites with significant contribution to the discriminations between inoculated and control plants based on OPLS‐DA. [file PBI-14-1589-s004.doc]

**Table S3:** Metabolites with significant contribution to the discriminations between inoculated and control plants based on OPLS-DA.

| **Keys** | **Metabolites** | | **Changes in resistant (DV)**  **inoculated vs control** | | | | | **Changes in susceptible (JG)**  **inoculated vs control** | | | | | |
| --- | --- | --- | --- | --- | --- | --- | --- | --- | --- | --- | --- | --- | --- |
|  | |  | | **2 DAI** | **4 DAI** | **8 DAI** | **12 DAI** | | **2 DAI** | **4 DAI** | **8 DAI** | **12 DAI** |  |
| 11 | | Acetate | | - | - | - | -0.88 | | - | - | - | - |  |
| 50 | | Adenosine | | - | - | - | - | | - | - | - | 0.91 |  |
| 9 | | Alanine | | - | -0.77 | - | - | | - | -0.92 | -0.74 | 0.84 |  |
| 16 | | Aspargine | | - | -0.64 | - | - | | -0.69 | -0.78 | - | 0.98 |  |
| 1 | | Cholesterol | | - | - | 0.71 | 0.93 | | - | - | 0.61 | - |  |
| 41 | | Clotrimazole | | - | - | - | 0.71 | | 0.65 | 0.69 | - | -0.69 |  |
| 27 | | Fructose | | - | - | - | -0.66 | | -0.94 | - | - | -1.00 |  |
| 36 | | Genistein | | - | 0.93 | 0.98 | 0.99 | | 0.98 | 0.98 | 0.98 | - |  |
| 22,23 | | Glucose | | -0.62 | - | - | - | | - | - | - | - |  |
| 12 | | Glutamate | | 0.81 | 0.78 | - | - | | 0.78 | 0.81 | 0.75 | 0.86 |  |
| 13 | | Glutamine | | 0.83 | 0.80 | - | - | | 0.81 | 0.83 | 0.64 | 0.91 |  |
| 46 | | Guanidoacetate | | - | - | - | - | | 0.96 | 0.70 | - | - |  |
| 51 | | Inosine | | 0.75 | - | - | - | | - | 0.76 | - | -0.74 |  |
| 2 | | Isoleucine | | - | - | - | - | | -0.80 | - | - | - |  |
| 3 | | Leucine | | - | - | - | - | | -0.79 | - | - | - |  |
| 6 | | Lipid | | - | - | - | - | | - | 0.87 | - | 0.98 |  |
| 38 | | Luteolin | | - | - | - | - | | - | 0.82 | - | -0.66 |  |
| 10 | | Lysine | | -0.78 | - | - | - | | -0.78 | - | - | - |  |
| 25 | | Malate | | - | -0.82 | -0.92 | -0.67 | | - | 0.97 | -0.97 | -0.92 |  |
| 39 | | Orotate | | 0.88 | 0.91 | 0.95 | 0.94 | | 0.83 | 0.96 | 0.88 | - |  |
| 47 | | Quinone | | 0.95 | - | 0.98 | 0.97 | | - | - | 0.97 | - |  |
| 21 | | Succinate | | - | - | - | - | | - | - | - | - |  |
| 26 | | Sucrose | | -0.63 | - | - | -0.78 | | -0.95 | -0.89 | - | -1.00 |  |
| 8 | | Threonine | | -0.67 | - | - | - | | -0.86 | -0.93 | -0.92 | 0.99 |  |
| 53 | | TMAO | | - | - | - | - | | 0.82 | - | - | - |  |
| 43 | | Tryptophan | | - | - | - | - | | - | - | - | 0.99 |  |
| 30 | | Uridine | | 0.98 | 0.95 | 0.98 | 0.98 | | 0.96 | 0.99 | 0.99 | 0.82 |  |
| 4 | | Valine | | - | - | - | - | | -0.72 | -0.87 | -0.70 | 0.88 |  |
| 48 | | 4-Nitrophenol | | - | - | - | 0.71 | | 0.67 | 0.77 | - | -0.65 |  |
| 32 | | 1-Unknown | | -0.80 | - | - | - | | -0.88 | -0.85 | -0.68 | - |  |
| 33 | | 2-Unknown | | -0.71 | - | - | - | | -0.82 | -0.86 | -0.69 | - |  |
| 35 | | 3-Unknown | | -0.70 | - | - | 0.71 | | 0.73 | - | - | - |  |
| 44 | | 4-Unknown | | - | - | - | - | | 0.84 | - | - | - |  |

The coefficient of 0.6 was used as a cutoff value for the significant difference evaluation (*P*< 0.05).

Positive values: up-regulated metabolites in inoculated with respect to control

Negative values: down-regulated metabolites in inoculated with respect to control; -: No significant change observed

Metabolites marked in red were confirmed by running their respective standards
