# Supplementary material for: Fusarium oxysporum mediates systems metabolic reprogramming of chickpea roots as revealed by a combination of proteomics and metabolomics
Source: Plant Biotechnol J. 2016 Jan 23;14(7):1589–603. doi: 10.1111/pbi.12522 (PMC5066658; doi:10.1111/pbi.12522)
Supplement: Supplementary file 9 — Table S4 List of primers used in quantitative real‐time PCR. [file PBI-14-1589-s010.doc]

**Table S4: List of primers used in quantitative real-time PCR**

| **Accession** | **Gene name** | **Primer(5’ 3’)** | **Product Length (bp)** |
| --- | --- | --- | --- |
| XM_004498429 | Glutamate synthase | F: TGATGGGGAGGAGGACTGA  R: GGGAGCAAAACCTGGTGAAG | 115 |
| XM_004512714 | Glutamate dehydrogenase | F: CAAATCACCCAACTGACCCA  R: GCTAACTGTAACACCTCCTG | 101 |
| XM_004490556 | Asparagine synthetase | F: TGGTGTCAAATGGGTAATCTCTG  R: TTCTCTGTGAAACTCTTCCTTGTC | 100 |
| XM_004512714 | Glutamine synthetase | F: AGTCACCCAGATGTTGTTGC  R: CCAGGAAAGCCACCAATAGG | 113 |
| CAA10131.1 | Chalcone synthase | F: TATGTCAAGTGCGTGTGTATTGTTT  R: CTCCTGTTGTCTTCAGTCCATCTTT | 80 |
| XP_004497326.1 | Chalcone isomerase | F: GTTGCTCCAGACCCTTGATTTCTAC  R: TGCCACACAATTCTCCATTACCTTC | 130 |
| XP_004505108.1 | Isoflavanoid synthase | F: GGGTCTTGTTGTGGATTTCTTCTCT  R: GCTTTCTTCATCACTCTTGGGTTGT | 105 |
| NP_001266030.1 | Isoflavone reductase | F: TACCATAAGAGCAGCAAATGACCC  R: TTTCTCCCACAAGGATACAACTTCA | 107 |
| XP_004505107.1 | Isoflavone 4'-O-methyltransferase | F: CATCCTCCATCGTTTCTTACGCC  R: TGCTATTTCTCCTTCTCCACCTTCT | 94 |
| XP_004505242.1 | Caffeoyl-CoA O-methyltransferase | F: CACCTCCTGATGCTCCTCTC  R: CGACGGCAGATAGTGATTCCA | 139 |
| XP_004501704.1 | Nuclear factor Y subunit C-1 | F: AACTCACCATTCGTTCTTGGCTT  R: TATCAGTCCTCGTAATAGCAGCAG | 93 |
| XM_004512108.1 | SKP1-like protein 1A | F: TCGCAGACAATCAAGCACAT  R: TAGGTTTATCATCAGAGGTCGCAG | 96 |
| NM_001282317.1 | AdoMet synthetase | F: AAGTTTCTTATGCTATTGGTGTTCC  R: CTTATCAGGTATCTTTCCAGTTCCA | 80 |
| NM_001279076.1 | Methionine synthase | F: GCTGGAGTGGTTGATGGAAGGA  R: AGTGTGAAGAAGTGAGCAGGAGG | 132 |
| XM_004513381 | Eukaryotic initiation factor 4A | F: TGTGCTAGATGAGGCTGATG  R: GCAGAGAAAACTCCCACTTG | 105 |
