# Supplementary material for: Fusarium oxysporum mediates systems metabolic reprogramming of chickpea roots as revealed by a combination of proteomics and metabolomics
Source: Plant Biotechnol J. 2016 Jan 23;14(7):1589–603. doi: 10.1111/pbi.12522 (PMC5066658; doi:10.1111/pbi.12522)
Supplement: Supplementary file 10 — Supplementary Legends [file PBI-14-1589-s006.doc]

# *Fusarium oxysporum* mediates systems metabolic reprogramming of chickpea roots as revealed by a combination of proteomics and metabolomics

# Yashwant Kumar#†, Limin Zhang#‡, Priyabrata Panigrahi†, Bhushan B. Dholakia†, Veena Dewangan†, Sachin G. Chavan†, Shrikant M. Kunjir€, Xiangyu Wu‡, Ning Li‡, Pattuparambil R. Rajmohanan€, Narendra Y. Kadoo†, Ashok P. Giri†, Huiru Tang‡§*, Vidya S. Gupta†*

# SUPPLEMENTAL MATERIALS

**Figures Legends:**

**Figure S1:** The Foc resistant-DV and susceptible-JG62 (JG) chickpea plants at early and late stages after inoculation. The letters ‘C’ or ‘I’ after the names of the cultivars indicate Control (mock-inoculation) or Foc inoculation, respectively, while the numbers 2 or 12 indicate the number of days after inoculation.

**Figure S2:** Permutation test results for OPLS-DA models with two components and 200 permutations. Models of (a-d) resistant (DV) chickpea plants are shown at 2, 4, 8 and 12 DAI and (*e-h*) susceptible (JG62) of same stages. a: DVC2 vs. DVI2 (intercepts: R^2^=0.0, 0.626; Q^2^=0.0, -0.196); b*:* DVC4 vs. DVI4 (intercepts: R^2^=0.0, 0.742; Q^2^=0.0, -0.254); c: DVC8 vs. DVI8 (intercepts: R^2^=0.0, 0.694; Q^2^=0.0, -0.0844); d: DVC12 vs. DVI12 (intercepts: R^2^=0.0, 0.683; Q^2^=0.0, -0.0642); e: JGC2 vs. JGI2 (intercepts: R^2^=0.0, 0.63, Q^2^=0.0, -0.199); f: JGC4 vs. JGI4 (intercepts: R^2^=0.0, 0.6; Q^2^=0.0, -0.0601); g: JGC8 vs. JGI8 (intercepts: R^2^=0.0, 0.651, Q^2^=0.0, -0.132) and h: JGC12 vs. JGI12 (intercepts: R^2^=0.0, 0.574, Q^2^=0.0, -0.0575).

**Figure S3:** Pattern of lignification in cross sections of chickpea root tissue from resistant-DV and susceptible-JG62 at different Foc inoculation stages using phloroglucinol/ hydrochloric acid stain

**Figure S4:** Design of experiment with details of sample preparation, tissue collection stages, approaches and data analysis.

**Figure S5:** Protein quality control measurements. (a) PCA plot indicating clear separation between control and inoculated samples; (b) reproducibility of intensity in replicates of samples; (c) majority of ion counts with less than 3 ppm error; and (d) percent coefficient of variance of retention time (% CV RT).

**Tables:**

**Table S1:** *A*, List of all the identified proteins using LC-MS^E^ with their details; *B***,** List of 481 differentially accumulated proteins identified using LC-MS^E^ with their details; *C*, List of 481 differentially accumulated proteins with their fold changes

**Table S2**: Assignments of metabolites from ^1^H-NMR analysis

**Table S3:** Metabolites with significant contribution to the discriminations between inoculated and control plants based on OPLS-DA.

**Table S4**: List of primers used in quantitative real-time PCR
